# Effect of different modification by gold nanoparticles on the electrochemical performance of screen-printed sensors with boron-doped diamond electrode

**DOI:** 10.1038/s41598-023-48834-7

**Published:** 2023-12-06

**Authors:** Oleksandr Matvieiev, Renáta Šelešovská, Marián Marton, Michal Hatala, Radovan Metelka, Martin Weis, Marian Vojs

**Affiliations:** 1 Faculty of Chemical Technology, Institute of Environmental and Chemical Engineering, University of Pardubice, Studentská 573, 532 10 Pardubice, Czech Republic; 2 Faculty of Electrical Engineering and Information Technology, Institute of Electronics and Photonics, Slovak University of Technology in Bratislava, Ilkovičova 3, Bratislava, 812 19 Slovak Republic; 3 Department of Graphic Arts Technology and Applied Photochemistry, Faculty of Chemical and Food Technology, Slovak University of Technology in Bratislava, Radlinského 9, Bratislava, 812 37 Slovak Republic; 4 Department of Analytical Chemistry, Faculty of Chemical Technology, University of Pardubice, Studentská 573, 532 10 Pardubice, Czech Republic

**Keywords:** Analytical chemistry, Sensors, Electrochemistry

## Abstract

Screen-printed sensors with chemically deposited boron-doped diamond electrodes (BDDE) were modified with different types of gold nanoparticles (AuNPs) according to a new original procedure. Physically and electrochemically deposited AuNPs had various sizes and also nanoporous character. They also differ in shape and density of surface coverage. The developed sensors were characterized using scanning electron microscopy and Raman spectroscopy. Their electrochemical properties were studied using cyclic voltammetry and electrochemical impedance spectrometry of selected *outer sphere* ([Ru(NH_3_)_6_]Cl_3_) and *inner sphere* (K_3_[Fe(CN)_6_], dopamine) redox markers. The application possibilities of such novel screen-printed sensors with BDDE modified by AuNPs were verified in the analysis of the neurotransmitter dopamine. The best analytical performance was achieved using printed sensors modified with the smallest AuNPs. The achieved limit of detection values in nanomolar concentrations (2.5 nmol L^−1^) are much lower than those of unmodified electrodes, which confirms the significant catalytic effects of gold nanoparticles on the surface of the working electrode. Sensors with the best electrochemical properties were successfully applied in the analysis of a model solution and spiked urine samples.

## Introduction

The development of new systems for the analysis of biologically active substances and their metabolites, important for the medical, agricultural, ecological, or food industry, is one of the most important areas of modern analytical chemistry^1,2^. Given that most of these substances are electrochemically active compounds, electrochemical methods are a great alternative to spectroscopic and separation methods of analysis^3,4^. Their main advantages are cheaper technology, low operating costs, ease of operation, portability, and easy miniaturization. Therefore, electrochemical methods can be used for analyses in the field or so-called point-of-care testing (POCT), for example, directly in doctors’ offices during therapeutic monitoring of the level of drugs and their metabolites in the blood/urine of patients^5,6^. This term generally includes the possibility of analysis at the point of sample collection without transporting to laboratories (especially in medicine, toxicology, environmental protection, etc*.*). These methods provide results very quickly and from a very small sample volume^3^. Therefore, it can be desirable to develop simple sensors and methods that do not require the work of a qualified analytical chemist.

Here, the trend toward miniaturization and simplification of electrochemical analyzes led to the creation of printed sensors, where the working electrode is based on boron-doped diamond. Boron-doped diamond electrode (BDDE) has been widely used due to its unique advantages: wide usable potential window, chemical resistance, resistance to passivation, low current noise, low background current, high hardness, and non-toxicity^7–9^. The application of BDDE to a screen-printed sensor in a novel way, using the chemical vapor deposition (CVD) method, enables superior stability of the sensor properties and long-term repeatability in thousands of measurements. In our previous work, we have already investigated the electrochemical properties and the possibilities of application of such sensors^10,11^.

Currently, the BDDE has many applications in electroanalytical chemistry for the analysis of drugs, pesticides, and other electrochemically active substances^1,12,13^. In recent years, however, there has been a trend to improve the properties of boron-doped diamond electrodes to meet the requirements of specific analytes, e.g., increasing the active area and improving selectivity and sensitivity^14^. This may lead to the application of BDDE within the aforementioned portable analyzers, POCTs and simple monitoring systems in the context of environmental quality, diagnosis of serious diseases or medical care and patient monitoring. For these reasons, surface modification with metal nanoparticles, metal oxides, carbon materials, organic molecules, and biomolecules was introduced^14^. Gold^15,16^, and others metals^17–19^ are usually used to modify the electrode surface with metal nanoparticles.

Among those, modification of the BDDE electrode surface with gold nanoparticles (AuNPs) leads to improvement of its electrochemical properties, e.g., enhancing the reversibility of the electrode reaction, changing the electron transfer resistance, increasing the sensitivity, selectivity, etc. Furthermore, gold has a relatively high positive potential of electrochemical oxidation at *ca.* 1.5 V versus Ag/AgCl reference electrode, making it a good choice to couple it with BDDE to retain most of the wide electrochemical window of the diamond^20^.

Several methods are used to modify the surface of the BDDE with nanoparticles. The first method is the formation of a suspension of nanoparticles (NPs) in a solution, followed by its application to the electrode surface and drying^21^. However, this method does not ensure excellent adhesion of NPs on the surface of the electrode and has low reproducibility. The second method is the physical deposition of AuNPs on the surface of the electrode under vacuum^21^. In such a way, a very small amount of metal is applied to form NPs, or a certain layer of metal is applied with a subsequent step of nanoparticle formation. The third method of applying AuNPs utilizes electrochemical deposition. This method is more widely used due to the variety of electrodes and methods of electrodeposition^22^. At the same time, AuNPs can be formed from various environments containing chlorides, sulfates, complexes, and many other electrolytes. As a method of application, constant potential or current, pulse, and other ways of electrodeposition can be used. Because the size, shape, and preferential crystallographic orientations of AuNPs strongly affect their catalytic properties^23^, the methods of preparation and the resulting geometry on the surface of BDDE should be thoroughly investigated. The size of gold nanoparticles plays a pivotal role in determining the catalytic activity in oxygen reduction^24^, and gold with (111) crystallographic facets exhibits higher electrocatalytic activity than (100) facets when it comes to electrochemical hydrogen evolution^25^.

Therefore, this work is devoted to develop promising sensors with BDDE modified with different types of gold nanoparticles prepared by various methods. Physical vapor and electrochemical depositions were used to form nanoparticles on the surface of BDDE. The analytical performance of BDDE with nanoporous AuNPs fabricated using a de-alloying method is evaluated for the first time. The effect of the size of the AuNPs and the deposition method on the sensors' electrochemical properties is studied. The possibility of application of proposed screen-printed sensors with a chemically deposited BDDE modified with AuNPs is tested for the determination of dopamine (DA). Its accurate and rapid determination is key to diagnosing many diseases^26^. The electrochemical behavior of DA is well researched and described in the literature^27^, so it was possible to objectively compare the results obtained using the new modified sensors presented in this work with those from the literature for unmodified BDDE or other modified electrode types.

## Results and discussion

### Material characterization

**Figure 1:**
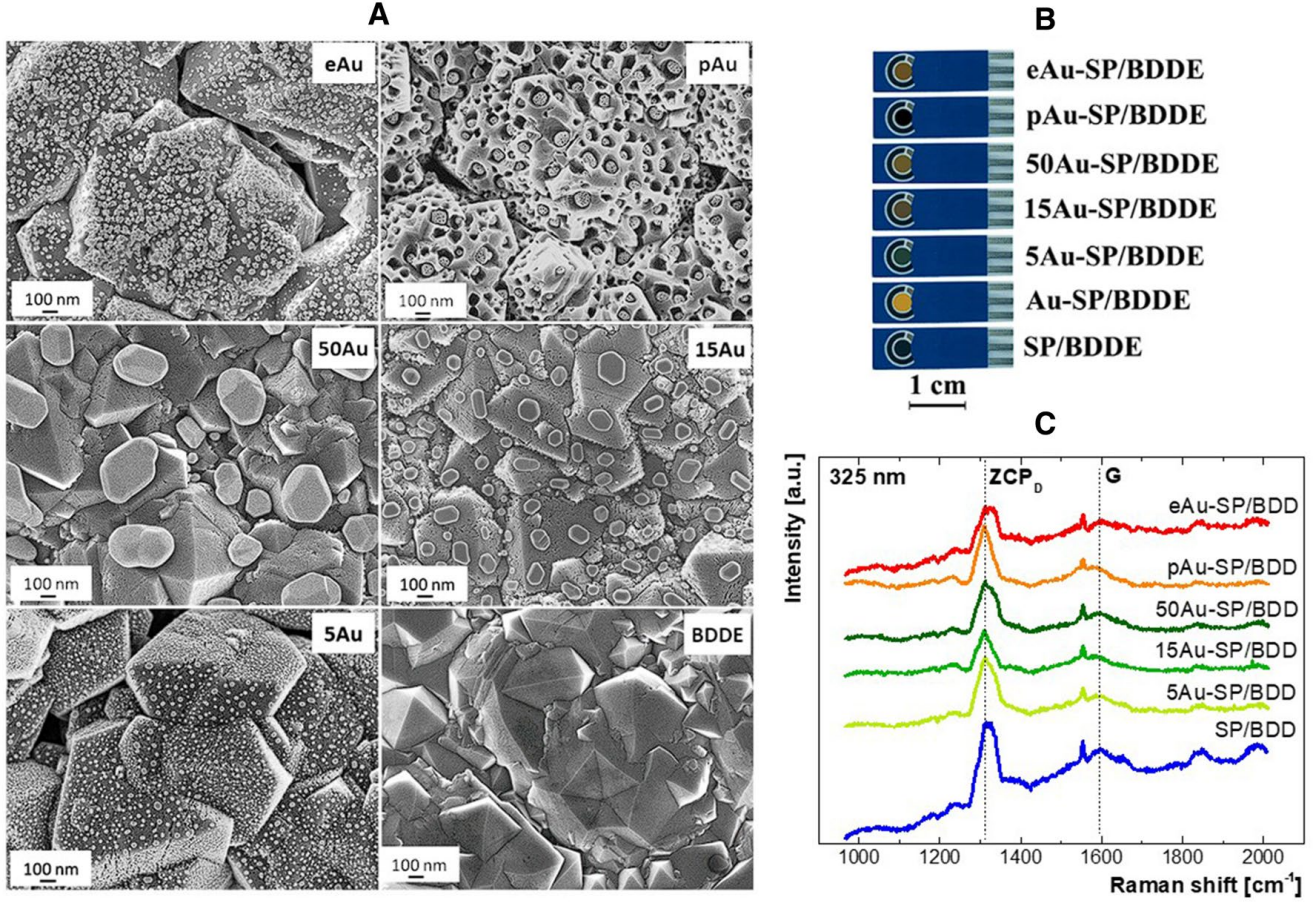
SEM micrographs (**A**), optical photographs (**B**), and UV Raman spectra (**C**) of as-grown BDDE and BDDE modified with AuNPs (ZCP_D_—zone center phonon mode of cubic diamond, G band—graphite band related to sp^2^ carbon).

Scanning electron microscopy (SEM) observations show homogeneous coverage of the BDDE surface by Au nanoparticles produced using both electrochemical and physical methods (Fig. 1A). The size of the physically prepared NPs depends on the thickness of the deposited Au layer; the thicker the Au layer, the larger NPs formed during the thermal annealing on the BDDE surface. The different sizes of formed NPs or the method of their production also caused a color change of the working electrode (WE), which is visible by naked eye (Fig. 1B) and suggest that the NPs obtained by the two presented methods cover the entire surface of the WE evenly. The highest density was observed for NPs formed by electrochemical means, which also differ in shape from those obtained by the physical deposition, being rougher, somewhat resembling “stars” (Fig. S1 in Supplementary Materials (SM)).

Examination of the size distribution of NPs using static histograms (Fig. S2) shows the smallest size of NPs obtained on the 5Au-SP(screen-printed)/BDDE sensor (applied Au layer of the thinnest thickness (*n* = 5 nm) and subsequent annealing), where it reached size of 16.8 ± 1.7 nm. In the case of applying a 15 nm layer of gold, two sizes of NPs are present, a much larger amount with a smaller size of 25.1 ± 0.8 nm and a smaller amount with a larger size of 128.1 ± 5.1 nm. In the case of the 50Au-SP/BDDE sensor, NPs with a size of 317.4 ± 24.4 nm were obtained. When producing the pAu-SP/BDDE sensor (see Fabrication of pAuNPs in SM for more details, Fig. S3), AuNPs with a size of 54.1 ± 1.6 nm were obtained, in which there are pores with a size of 6.9 ± 0.2 nm. After electrochemical deposition, nanoparticles of the size of 21.7 ± 0.9 nm were obtained. Tiny dimples are also visible on the surface of the diamond crystals due to thermal annealing in N_2_ atmosphere, which also caused the porous AuNPs to be embedded to a shallow depth, presumably due to a catalytic reaction of the Au/Ag compound (more details in Supplementary Material in “Fabrication of pAuNPs”). Such ‘anchoring’ of gold nanoparticles can have a positive effect on the long-term stability of the electrode.

To examine the chemical composition of BDDE modified by Au and compare it with the as-grown BDD, UV Raman spectroscopy was employed (Fig. 1C). Acquired spectra show the typical diamond maximum at approximately 1310 cm^−1^ (ZCP_D_) related to zone center phonon mode of cubic diamond, shifted from its original position at 1332.9 cm^−1^ due to the ‘Fano’ effect and phonon confinement caused by boron incorporation^28^. The broad maximum at approximately 1590 cm^−1^ (G) relates to the non-diamond carbon content, mostly sp^2^-bonded carbon^29^. The ratio of intensities of G and ZCP_D_ maxima (I(G)/I(ZCP_D_), Table S1) shows a deviation below 10% from the pristine BDD reference value. Therefore, it is evident that the modification with Au had no or negligible effect on the BDDE surface in terms of the non-diamond carbon phase content for all investigated electrodes.

### Electrochemical characterization

#### Cyclic voltammetry

The electrochemical properties of the tested sensors were studied using cyclic voltammetry (CV) by analyzing repeatability, reversibility, and reaction’s control. Three redox markers were employed to characterize the effect of different modifications by gold nanoparticles on the electrochemical behavior with *inner* and *outer sphere* nature of electron transfer (ET). The *inner sphere* ET makes [Fe(CN)_6_]^4−/3−^ and DA, unlike the *outer sphere* redox markers ([Ru(NH_3_)_6_]^2+/3+^), extremely dependent and sensitive to the surface chemistry of the electrodes. Very good electrochemical performance was found mainly for BDDE modified with the smallest NPs, indicating catalytic effect of AuNPs, which was most significant for the *inner sphere* redox markers.

[Ru(NH_3_)_6_]^2+/3+^ was used as an *outer sphere* redox marker, which is not sensitive to the electrode surface^30^. For these markers, the electrochemical reaction is not a limiting factor; i.e., the electron transfer reaction occurs at a very high rate, and reagents, intermediates, and reaction products are not adsorbed on the surface and do not interact with the double layer of the electrode^31^. Subsequently, two redox markers of the *inner sphere* ([Fe(CN)_6_]^4−/3−^ and DA), which are sensitive to the surface of the electrode, were also tested^32^. The electron transfer rate of *inner sphere* redox markers is strongly influenced by the character of the electrode surface, e.g., the presence of nanoparticles; and the involved reactants, intermediates, and products interact strongly with the double layer and the electrode surface^33^.

First, all electrodes showed a very good repeatability of the peak currents results (relative standard deviation, RSD_10_ < 4%), except for *inner sphere* redox markers on Au-SP/BDDE. This parameter was analyzed by measuring ten repeated cyclic voltammograms for all redox markers at *v* of 100 mV s^–1^ in the electrolyte of 0.1 M KCl for [Ru(NH_3_)_6_]^2+/3+^ and [Fe(CN)_6_]^4−/3−^, and in Britton–Robinson buffer (BRB, pH 5.5) for DA. The results obtained from these measurements are shown in Fig. S4 for [Ru(NH_3_)_6_]^2+/3+^, Fig. S5 for [Fe(CN)_6_]^4−/3−^ and Fig. S6 for DA.

Next, the reversibility of the ongoing electrochemical reactions characterized by the ratio of anodic and cathodic peak heights (*I*_pa_/*I*_pc_) and the potential difference between the anodic and cathodic peaks (Δ*E*_p_) showed a reversible behavior for all types of electrode modifications (except for the Au-SP/BDD with a quasi-reversible behavior) using the *outer sphere* redox marker. However, using the *inner sphere* markers the results were strongly affected by the surface modification, showing the best results for the BDDE with electrochemically deposited NPs and for the BDDE with the smallest NPs produced by the physical method, worsened with increasing NPs size.

**Figure 2:**
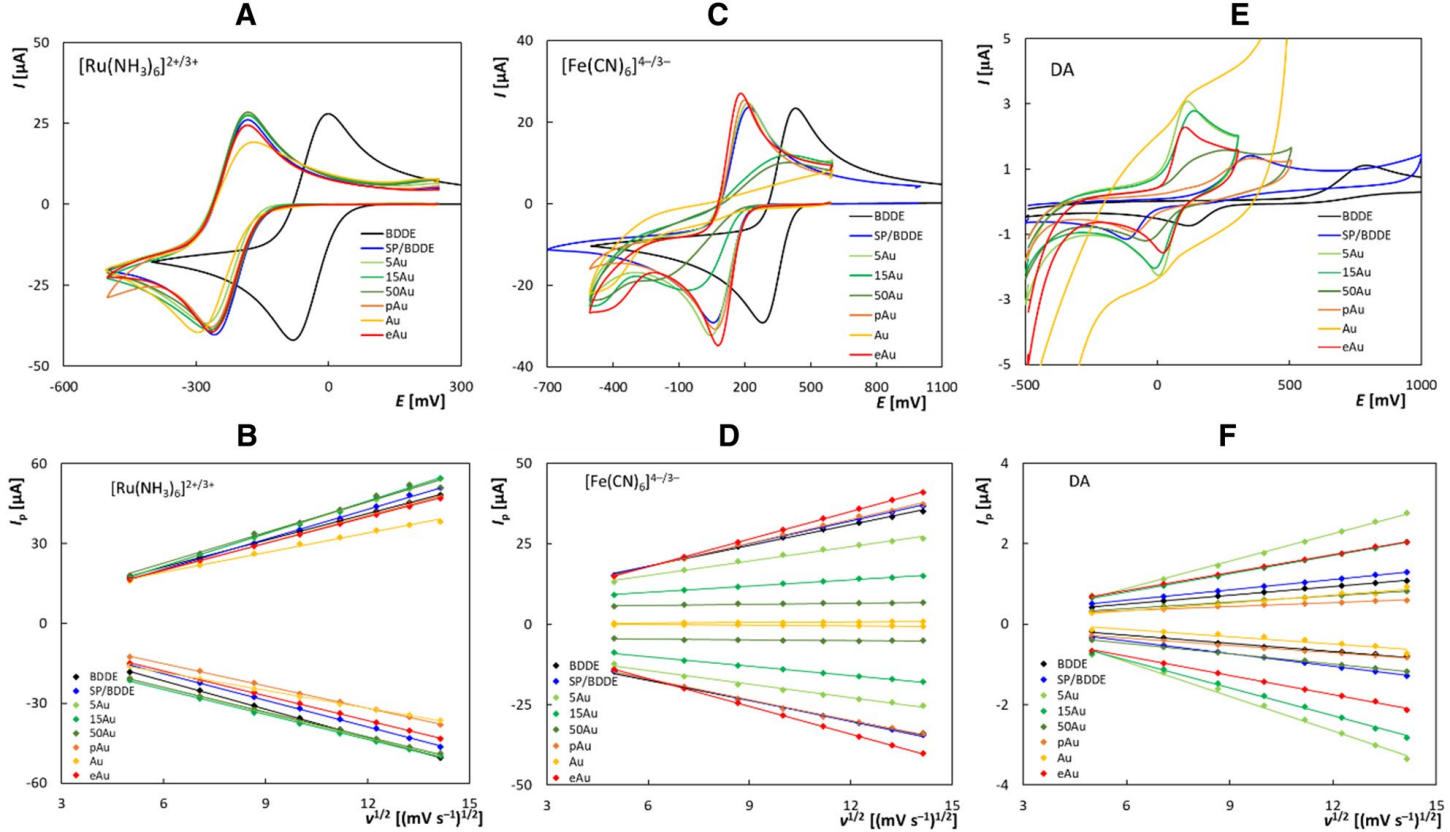
Cyclic voltammograms of [Ru(NH_3_)_6_]^2+/3+^ (**A**), [Fe(CN)_6_]^4−/3−^ (**C**), and DA (**E**) together with dependences of *I*_p_ on the *v*^1/2^ obtained for [Ru(NH_3_)_6_]^2+/3+^ (**B**), [Fe(CN)_6_]^4−/3−^ (**D**), and DA (**F**) using the tested sensors (electrolyte—0.1 mol L^−1^ KCl (**A**–**D**), BRB pH 5.5 (**E**, **F**), *v* = 100 mV s^−1^ (**A**, **C**, **E**), 25–200 mV s^−1^ (**B**, **D**, **F**), *c*([Fe(CN)_6_]^4−/3−^) = *c*([Ru(NH_3_)_6_]^2+/3+^) = 2.5 mmol L^−1^,* c*(DA) = 50 µmol L^−1^).

Figure 2A shows the cyclic voltammograms of [Ru(NH_3_)_6_]^2+/3+^ recorded on all tested electrodes. The particular values of anodic and cathodic peak heights (*I*_pa_ and *I*_pc_), their ratio (*I*_pa_/*I*_pc_), and the values of the anodic and cathodic peak potential (*E*_pa_ and *E*_pc_) and their difference (Δ*E*_p_) are summarized in Table S2. The obtained peak heights were quite similar, but the lowest value was in the case of Au-SP/BDDE. The *I*_pa_/*I*_pc_ parameter ranged from 0.9 to 1.3, which confirms the reversibility of the reaction. The potential difference between the anodic and cathodic peaks, which is an important criterion for evaluating the reversibility of the electrode reaction, was close to the theoretical value of 59 mV, except for the case of Au-SP/BDDE, where it was 110.8 mV. The significant shift of potentials to more negative values in the case of all types of lab-made SP/BDDE compared to the classic three-electrode set-up is due to the use of a silver pseudo-RE, which was confirmed in our previous work^10^.

A similar experiment for [Fe(CN)_6_]^4−/3−^ is shown in Fig. 2C, and the parameters of the measured voltammograms are summarized in Table S2. In case of this redox probe, a more interesting situation was observed. The *I*_pa_/*I*_pc_ parameter was also in the range of 0.9 to 1.3 on all sensors tested, which is very close to the theoretical value of 1 and confirms the reversibility of the electrode reaction. The highest peaks and the best reversibility were obtained on eAu-SP/BDDE. On the other hand, small oxidation and reduction peaks were documented on Au-SP/BDDE. In the case of using BDDE, SP/BDDE, and pAu-SP/BDDE, similar but worse results were obtained in comparison to those acquired with the electrochemically modified electrode. A very interesting dependence was observed in the case of 5Au-SP/BDDE, 15Au-SP/BDDE, and 50Au-SP/BDDE, where the *I*_p_ and the reversibility of the reaction worsened with an increase in the size of the AuNPs.

The recorded cyclic voltammograms for DA are shown in Fig. 2E, and the statistical parameters are summarized in Table S2. The *I*_pa_/*I*_pc_ parameter was in the range of 0.7 (50Au-SP/BDDE) to 1.4 (BDDE) at all sensors tested. Again, small oxidation and reduction peaks were observed in the case of Au-SP/BDDE. The highest peaks were obtained using 15Au-SP/BDDE, 5Au-SP/BDDE, and eAu-SP/BDDE. The lowest Δ*E*_p_ value (80 mV) and thus the best reversibility of the ongoing reaction was recorded for eAu-SP/BDDE. For *n*Au-SP/BDDEs, this difference was slightly larger and increased with increasing nanoparticle size, which may indicate a slower electrode reaction. Several times higher Δ*E*_p_ was observed for SP/BDDE and pAu-SP/BDDE, and the worst result was obtained for BDDE. Presumably, this is due to the catalytic effect of gold nanoparticles^34,35^.

The electrode reaction’s was kinetic-diffusion controlled in the case of [Fe(CN)_6_]^4−/3−^ and diffusion controlled in the case of [Ru(NH_3_)_6_]^2+/3+^ and DA, which was determined by investigating the influence of the *v* on the voltammograms of these redox markers. This experiment was carried out in the *v* range of 25–200 mV s^–1^, and the measured voltammograms are shown in Fig. S7 (for [Ru(NH_3_)_6_]^2+/3+^), Fig. S8 (for [Fe(CN)_6_]^4−/3−^), and Fig. S9 (for DA). The *I*_p_ of all redox markers increased with increasing *v*, but the corresponding dependences were nonlinear. Instead, linear dependences were observed between the *I*_p_ and the *v*^1/2^, which are shown in Fig. 2B (for [Ru(NH_3_)_6_]^2+/3+^), Fig. 2D (for [Fe(CN)_6_]^4−/3−^), and Fig. 2F (for DA). Statistical parameters of these dependencies are summarized in Table S3. Statistical parameters of the logarithmic dependences of *I*_p_ on *v* are summarized in Table S4. In the case of [Fe(CN)_6_]^4−/3−^ redox marker measured on all electrodes, the slope was less than 0.5, indicating the electrode reaction's kinetic-diffusion control^36^. In the case of the DA redox marker, the slope for all sensors was greater than 0.5, indicating the diffusion-controlled electrode reaction with the slight influence of adsorption^36^.

#### Electrochemical impedance spectroscopy

The electrochemical properties of the tested sensors were further studied using electrochemical impedance spectroscopy (EIS) with all three redox markers. The investigation revealed decreased electron transfer resistance and increased apparent heterogeneous electron transfer rate constant, i.e., accelerated electrochemical reactions, for BDDE modified with gold nanoparticles compared to the unmodified diamond and continuous gold layer. The sensor modified by electrochemically deposited gold nanoparticles (eAu-SP/BDD) showed the best value of electron transfer resistance, and for the physically deposited AuNPs, the electron transfer rate was clearly correlated with the NPs size, with smaller NPs yielding better performance.

**Figure 3:**
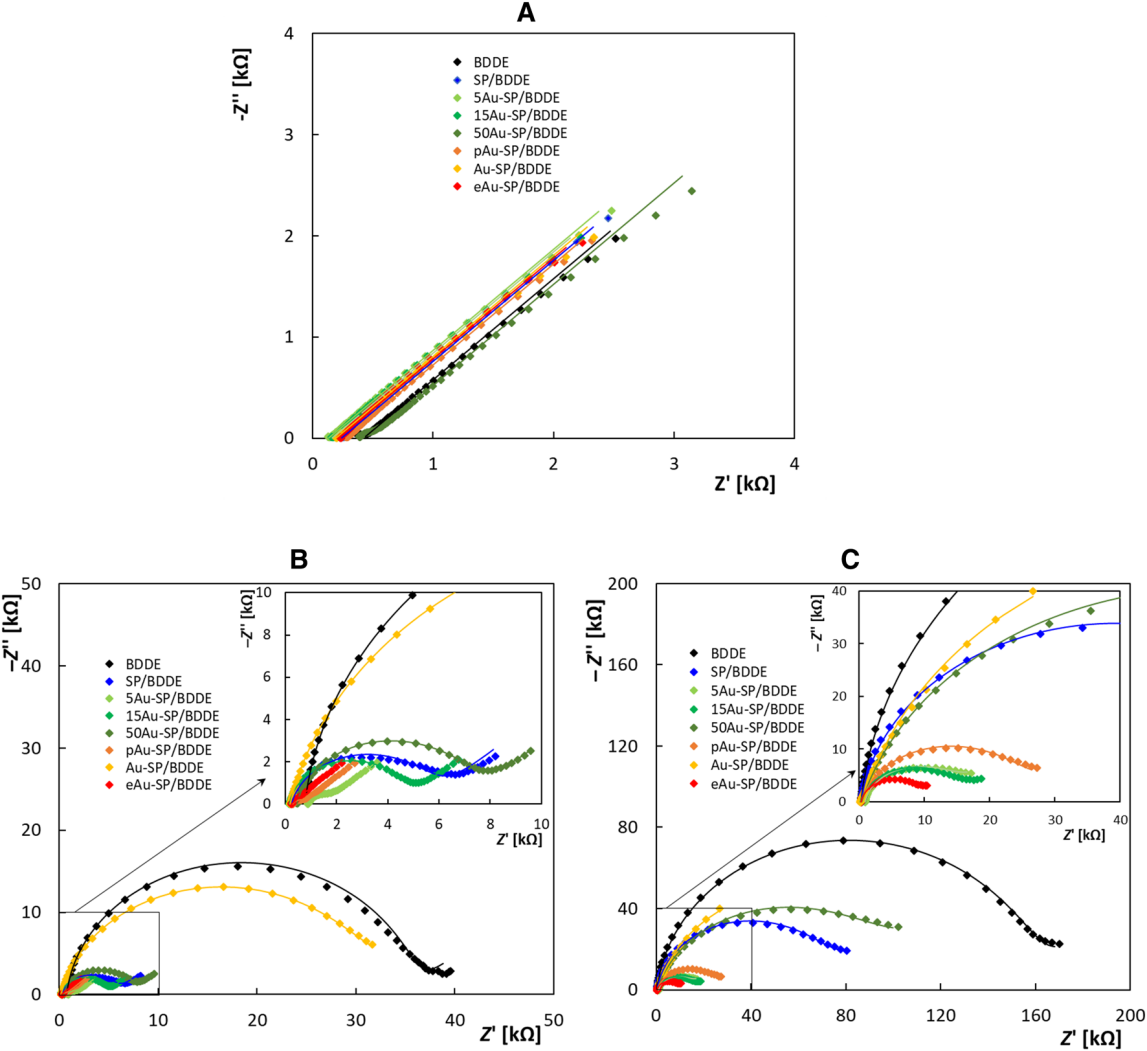
Electrochemical impedance spectra (Nyquist plots) recorded on the tested electrodes in 2.5 mmol L^−1^ [Ru(NH_3_)_6_]^2+/3+^ (**A**), 2.5 mmol L^−1^ [Fe(CN)_6_]^4−/3−^ (**B**), and 2.5 mmol L^−1^ DA (**C**) in 0.1 mol L^−1^ KCl (*f* = 0.1–100 k Hz, *A* = 10 mV).

In the case of the EIS investigation of the redox marker [Ru(NH_3_)_6_]^2+/3+^ (Fig. 3A), a straight line at an angle very close to 45° was observed in Nyquist plots, i.e., it corresponds to Warburg's impedance (diffusion impedance)^37^. Each measured value was calculated as the average of 3 repeated measurements. The Bode impedance plots and the Bode phase plots are shown in Fig. S10A, B. This was an expected fact since this redox marker was shown to be characterized as an *outer sphere* redox marker that is not sensitive to the surface of the electrode.

All the calculated quantities^38^ are summarized in Table S5. The electron transfer resistance and the electron transfer rate calculated from it fluctuated in a wide range. This is primarily related to the specifics of the electrochemistry of the redox marker. The results show that the Warburg coefficients and the diffusion coefficients are very close, indicating that the diffusion takes place on all unmodified and modified electrodes based on the same mechanism and at a relatively close rate.

As for other so-called *inner sphere* redox markers, such as [Fe(CN)_6_]^4−/3−^ and DA, they are very sensitive to the surface of the electrode. The results of the impedance spectra (see Fig. 3B,C) gave insight into the effect of the modification on its electron transfer resistance. The Bode impedance plots and the Bode phase plots are shown in Fig. S10C–F. An electrical circuit *R*(*Q*/[*RW*]) was used to fitting and simulation these redox markers. A constant phase element (*Q*) was used to calculate the double-layer capacitance at the surface of all unmodified and modified electrodes^20^, and all the calculated quantities are shown in Table S6.

The series resistance R_s_ (in case of “*inner sphere*” markers), which corresponds to the electrolyte resistance in the case of all electrodes, was in the range from 169 to 926 Ω. This is mostly related to the manufacturing procedure, as it is difficult to manufacture sensors with the same resistance due to the heterogeneity of the diamond polycrystals. A decrease in electron transfer resistance and an increase in apparent heterogeneous electron-transfer rate constants (*k*^0^_app_)^20^ were observed in the case of almost all modified electrodes, indicating an electrochemical reaction acceleration. Increasing the size of the nanoparticles fabricated by the physical deposition of gold leads to a decrease in the electron transfer rate. No dependence on the effect of electrode modification with gold nanoparticles on the diffusion or effective capacitive surface was found. On the other hand, the EIS is a powerful tool to investigate the analyte as well as electrode material properties^39^ if the appropriate equivalent electrical circuit model is selected. Note that the Maxwell–Wagner effect rules the charge relaxation processes on the analyte/electrode interface. As a result, the relaxation time (*τ*) given by the dielectric constant and conductivity ratio can be modelled utilizing the parallel resistor R and capacitor C^40^. Since the constant phase element (CPE) exponent approaches the value of 1, the CPE exhibits capacitance behavior, and one can approximately estimate the relaxation time as *τ* = *R*_ct_*C*_eff_. Interestingly, the BDDE exhibits greatly longest relaxation times, while SP/BDDE offers one order of magnitude faster relaxation. In addition, nanostructured surfaces, such as eAu-, 5Au- or 15Au- NPs or porous AuNPs exhibit the shortest relaxation time. It should be noted that the surface structures often affect surface diffusion^41^, which leads to the non-Cottrell behavior close to the patterned surface.

### Application of AuNPs modified BDDE in voltammetric determination of dopamine

Application of the modified electrodes for detection of DA revealed the highest sensitivity and lowest limit of detection (LOD) for the electrodes that showed the best overall performance in CV and EIS characterizations, i.e., the BDDE modified with the smallest AuNPs, regardless of the deposition method used. However, the surface morphologies of NPs (rough structure of eAu) and BDDE (pAu embedded in BDD), which were affected by the method of modification, influenced the accessibility of the electroactive centers, resulting in markedly different results at a lower scan rate.

The modified electrodes with the best performance were then applied in analysis of DA in model solutions and urine samples. By achieving correct and well-repeatable results (*RSD*_5_ < 5%), the developed sensors were proven not only in model solutions, but also in the analysis of biological samples with a more complex matrix.

#### Concentration dependences

Electrochemical oxidation of DA in an aqueous medium includes two two-electron oxidation processes. In the first stage, dopamine-o-quinone is formed, which can be reduced back to DA. The deprotonation of dopamine-o-quinone then leads to a cyclization reaction to form 5,6-dihydroxyindoline, which is more easily oxidized than DA, and again two-electron oxidation occurs to form aminochrome (Fig. S11).

**Figure 4:**
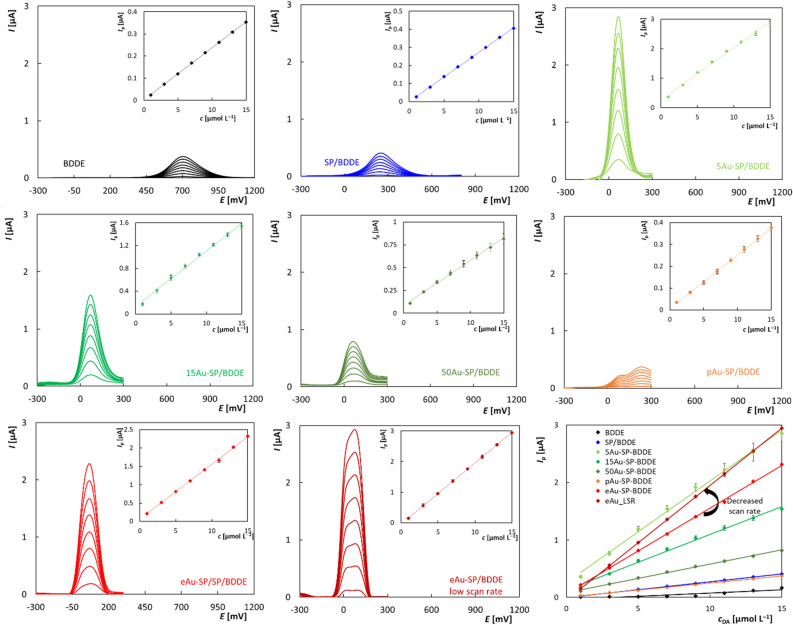
SW voltammograms of DA in dependence on concentration obtained for all tested sensors and the corresponding dependences of *I*_p_ on *c*; electrolyte—BRB (pH 5.5), *c*(DA) = 1–15 μmol L^−1^, ν = 50 mV s^–1^(and 5 mV s^–1^ for eAu-SP/BDDE), *A* = 80 mV, *f* = 10 Hz.

Developed SPE sensors with BDDE modified with AuNPs were used to measure the DA concentration dependencies using square wave voltammetry (SWV) with optimized parameters (see SM, “Optimization of the voltammetric analytical method”) in a given range (1–15 μmol L^−1^) to determine the basic statistical parameters. The recorded voltammograms with the appropriate dependences of *I*_p_ on *c*(DA) are shown in Fig. 4, which allows also a comparison of the dependencies obtained. The statistical parameters like slopes, intercepts, and correlation coefficients summarized in Table 1 indicate that the sensors with AuNPs generally increase the sensitivity of DA determination.

**Table 1:** Statistical parameters of peak height dependencies on DA concentration obtained on tested sensors; electrolyte—BRB (pH 5.5), *c*(DA) = 1–15 μmol L^–1^, *ν* = 50 mV s^–1^ (and 5 mV s^–1^ for eAu-SP/BDDE), *A* = 80 mV, *f* = 10 Hz.

Electrode	Slope [µA L µmol^−1^]	Intercept [µA]	*r*	LOD [nmol L^−1^]
BDDE	0.024 ± 0.001	0.001 ± 0.001	0.9998	62.2
SP/BDDE	0.027 ± 0.001	0.001 ± 0.001	0.9997	77.3
5Au-SP/BDDE	0.177 ± 0.004	0.258 ± 0.040	0.9998	2.5
15Au-SP/BDDE	0.097 ± 0.003	0.128 ± 0.025	0.9976	9.3
50Au-SP/BDDE	0.050 ± 0.001	0.082 ± 0.010	0.9998	36.6
pAu-SP/BDDE	0.025 ± 0.001	0.006 ± 0.002	0.9998	13.4
eAu-SP/BDDE	0.149 ± 0.002	0.064 ± 0.015	0.9997	12.8
eAu-SP/BDDE (5 mV s^–1^)	0.200 ± 0.001	− (0.044 ± 0.005)	0.9998	9.9

Of the sensors that were modified by physical vapor deposition, the NPs with the smallest size (5Au-SP/BDDE) showed the most significant increase in slope value (176.8 ± 4.3 nA L µmol^−1^) by 6.5 times compared to unmodified BDDE or SP/BDDE. Comparably excellent results were also obtained after the electrochemical deposition of the NPs (149.1 ± 1.6 nA L µmol^−1^). Decreasing the *v* on the eAu-SP/BDDE leads to a further increase in sensitivity (199.60 ± 0.54 nA μmol L^−1^). This can be explained by the fact that at a low *v*, all electroactive centers can be used, including those inside the structured material, which at higher speeds are not involved in ongoing redox reactions^42^. This effect was not observed for sensors with physically deposited NPs (except pAu-SP/BDDE), which the lower density of the electrode surface coverage with AuNPs can explain. In addition, the actual surface of the electrochemically deposited AuNPs is structured, while it is smooth in the case of physical vapor deposition (Fig. S1). In the case of pAuNPs, these are embedded deep in the diamond layer, which results in the involvement of the BDDE surface in the reactions, when the *v* is high, signalized by the rise of two oxidation peaks. After the *v* is slowed (Fig. S14), only the peak from reactions on pAuNPs is observed, interestingly with an unchanged high of *I*_*p*_. In contrast to eAu-SP/BDDE, sensitivity was not positively affected by decreasing the polarization rate in the case of pAu-SP/BDDE.

To calculate the LOD, the concentration dependences were also measured in the lower concentration range from 0.2 to 1 μmol L^−1^, which is shown in the supplementary material in Fig. S15. In the case of low *v* for eAu-AP/BDDE, two distinct oxidation peaks appear, which may be caused by the fact that during slow *v* it is most probably possible to distinguish two subsequent oxidation steps in electrochemical oxidation of DA. The calculated values of LOD are summarized in Table 1 and their comparison with other sensors described in the literature are shown in Table S7. It is obvious that the use of developed modified sensors leads to a decrease in the LOD. At the same time, it was found that the best results were achieved using sensors with smaller nanoparticles, such as 5Au-SP/BDDE and eAu-SP/BDDE (especially at low scan rates). Similar LOD values were also obtained for pAu-, although the sensitivity (24.5 ± 0.17) of these sensors was significantly lower. The LOD values achieved in this work for the newly designed AuNPs modified sensors are lower compared to those published in the literature for other types of gold modified sensors including BDDE (Table S7).

#### Determination of dopamine in model and real samples

The proposed method for the determination of dopamine using screen-printed sensors with BDDE modified with AuNPs was verified during the analysis of a model solution and a urine sample. Only the sensors with the smallest nanoparticles, which showed the best electrochemical properties in the previous chapters, were applied. The procedure for sample preparation and subsequent analysis is described in the "Materials and methods" chapter. Examples of these analyses, including a graphical evaluation of the standard addition method, are shown in Fig. S16 in the SM, and the obtained results are summarized in Table 2. It is clear that the tested sensors enable the achievement of correct and well-repeatable results (*RSD*_5_ < 5%) not only in model solutions, but also in the analysis of biological samples with a more complex matrix.

**Table 2:** Results of dopamine determination in model solution and urine sample.

Sample	Electrode	Added [µmol L^−1^]	Determined [µmol L^−1^]	Recovery [%]	*RSD*_5_ [%]
Model sample	5Au/SP-BDDE	1.0	(1.005 ± 0.045)	94.2–108.1	4.5
	eAu/SP/BDDE		(1.006 ± 0.024)	97.7–103.4	2.4
Urine	5Au/SP-BDDE	1.0	(0.995 ± 0.042)	95.1–104.2	4.8
	eAu/SP/BDDE		(0.998 ± 0.022)	96.3–104.5	3.9

## Conclusion

In this work, SPE with a chemically deposited BDDE were modified with AuNPs of different sizes and pAuNPs using physical and electrochemical deposition methods. It was confirmed that the modification of BDDE with AuNPs leads to the improvement of the electrochemical properties.

With the use of SEM, it was found that, depending on the increasing thickness of the physically applied Au film during the sensors' preparation, the resulting nanoparticles' size also increases. Compared to electrochemically applied NPs, which are rougher and somewhat resemble "stars", they have a different crystallographic structure, and their surface is smoother. The electrode surface coverage with NPs is homogeneous in all cases, but its density was the highest after electrochemical deposition.

The electrochemical properties of the tested sensors were studied applying CV of selected redox probes. The repeatability of the obtained cyclic voltammograms was excellent in all cases (RSD_10_ < 4%) with the exception of *inner sphere* redox markers on the sensor with the surface completely covered with gold. While in the case of the Ru complex, very similar results were recorded for all compared sensors, the catalytic effect of AuNPs on the electrode surface was significantly manifested in the case of *inner sphere* redox markers. The best results, the highest *I*_p_ and smallest Δ*E*_p_, were recorded for the smallest AuNPs, i.e., the eAu-SP/BDDE and 5Au-SP/BDDE. As the size of the NPs increases, the electrochemical properties, such as the rate of charge transfer, deteriorate. These results were confirmed also using EIS, when the modification with AuNPs leads to an acceleration of the electrochemical reaction compared to an unmodified diamond and pure Au. Moreover, the eAu-SP/BDDE showed the best value of electron transfer resistance.

The last part of the work was focused on testing the application possibilities of modified sensors in electroanalysis. DA was used as a model analyte to evaluate the analytical parameters of the tested sensors. Comparing the sensitivities of individual sensors, 5Au-SP/BDDE and then other sensors with a small size of nanoparticles yield the best results. The obtained LOD values in the order of units to tens of nmol L^−1^ are significantly lower than those of unmodified BDDEs as well as values previously published in the literature for the determination of DA using different AuNPs modified electrodes including BDDE.

## Material and methods

### Chemicals

A standard DA solution (0.001 mol L^–1^) was prepared by dissolution in distilled water in an ultrasonic bath. Due to the light instability of DA, the solution was stored in a refrigerator without access to light, and a new solution was prepared every 3 days. 0.1 mol L^–1^ KCl solution was used as the supporting electrolyte, prepared by dissolving solid potassium chloride in distilled water. Britton-Robinson buffer (BRB) was used in the pH range of 3–8. The acidic component of BRB was prepared by mixing H_3_PO_4_, H_3_BO_3_ and CH_3_COOH to a concentration of 0.04 mol L^–1^. The solution of NaOH (0.2 mol L^−1^) created the alkaline component. To study the electrochemical properties of the electrodes, solutions of potassium hexacyanoferrate (K_3_[Fe(CN)_6_]), hexaammineruthenium (III) chloride ([Ru(NH_3_)_6_]Cl_3_), and DA with a concentration of 2.5 mmol L^−1^ were prepared in 0.1 mol L^–1^ KCl, 50 µmol L^−1^ DA solution in BRB (pH 5.5).

Acetonitrile was used for cleaning the SPE and BDDE. A solution of HAuCl_4_∙4H_2_O with a concentration of 1 mmol L^–1^ in 0.1 mol L^–1^ H_2_SO_4_ was used for the electrodeposition of AuNPs. A KCN solution (0.1 mol L^–1^) was used to remove AuNPs.

### Electrochemical measurements

Electrochemical measurements were performed using Autolab PGSTAT204 (Metrohm Autolab, Utrecht, The Netherlands), equipped with Nova 2.1.5 software. In the case of the conventional three-electrode arrangement of the electrochemical cell, a working electrode (WE) from boron-doped diamond (BDDE, BioLogic, France, working area 7.07 mm^2^, B/C 1000 ppm), an auxiliary electrode (CE) from platinum wire, and a saturated silver/silver chloride (Ag/AgCl/KCl(sat.)) reference electrode (RE) (both Monokrystaly, Turnov) were applied. In addition, screen-printed sensors (SPE) with chemically deposited BDDEs as WE and CE and a quasi-RE from Ag/AgCl (SP/BDDE, WE surface area 7.07 mm^2^, the inner diameter of 3 mm, B/C 312 500 in the gas phase, resistivity of 0.017 Ω cm, *n* = 2.9 × 10^21^ cm^−3^)^43^ were used. Vacuum evaporation was used to modify the SPE with AuNPs of different sizes (*n*Au-SP/BDDE, where *n* = 5, 15 and 50 nm refers to the thickness of the gold layer applied by physical vapor deposition) and with nanoporous AuNPs (pAu-SP/BDDE). For comparison, SP/BDDE modified with AuNPs by electrochemical deposition (eAu-SP/BDDE) and SP/BDDE, in which the WE was completely covered with a layer of 100 nm Au using physical deposition, were also used.

### Preparation of screen-printed sensors with chemically deposited BDDE modified with AuNPs

**Figure 5:**
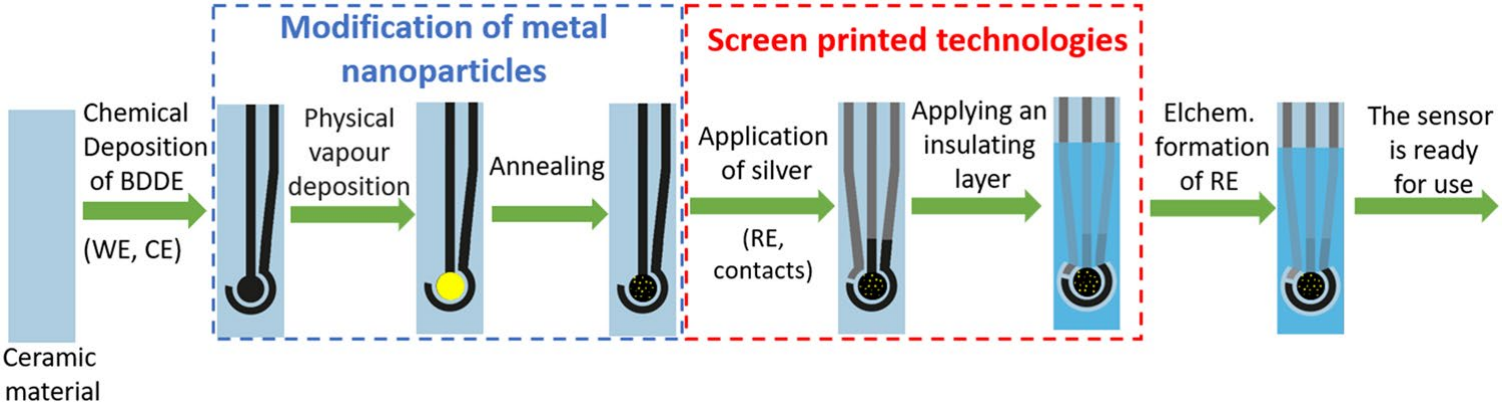
Preparation of the SPE with chemically deposited BDDE modified with AuNPs.

Modification of BDDE with Au and porous Au nanoparticles utilizing physical vapor deposition was carried out during the process of making the SPE described in our previous work^10,44^ in particular, after the step with CVD deposition of the BDDE layer (Fig. 5). The sensors, denoted as *n*Au-SP/BDDE, were modified by gold nanoparticles using thermal evaporation of Au in high vacuum on the surface of BDDE to form a layer of thickness *n* (*n* = 5, 15, and 50 nm) and subsequent annealing in a N_2_ atmosphere at 600 °C to dewet the Au layer and provide homogeneous dispersion and the final shape of AuNPs. Finally, porous AuNPs (pAu-SP/BDDE) were obtained by evaporating an Au/Ag bilayer, annealing to form an Au/Ag alloy (N_2_, 600 °C), and wet etching in hydrofluoric acid to remove Ag^45^ (see Fabrication of pAuNPs in SM for more details).

The electrochemical modification by AuNPs was performed after the complete fabrication of the unmodified SP/BDDE platform. An electrolyte containing 1 mmol L^–1^ HAuCl_4_∙4H_2_O in 0.1 mol L^–1^ H_2_SO_4_ was used for the electrochemical deposition of AuNPs. This composition of the electrolyte was chosen as a compromise of those available in the literature^16,22^. The potential and time of electrodeposition were optimized, and the optimal parameters for modification were 0 mV and 50 s, respectively.

Figure 1B shows the tested lab-made screen-printed (SP) electrodes with BDDE (SP/BDDE) modified with AuNPs and a bulk BDDE for a comparison.

### Surface characterization of tested sensors

A field emission scanning electron microscope (SEM, model JSM-7500F from JEOL Ltd., Tokio, JP) was used to examine the surface of the electrodes. Analysis of the size of AuNPs was carried out with the help of ImageJ software (from the National Institutes of Health and the Laboratory for Optical and Computational Instrumentation, University of Wisconsin). We selected a minimum of 50 NPs on each of the modified electrodes, and for each of them, we measured their area. Based on these areas, we calculated the diameter of each NP, which was then represented as a circle. The diameter distribution of nanoparticles on the surface of the electrode was analyzed using the Gaussian function in OriginPro 9.0 (OriginLab Corporation, Northampton, US). Raman spectra were obtained using a UV system equipped with a 325 nm laser excitation source (Spectroscopy&Imaging, Germany, 2 μm spot diameter). The 325 nm laser wavelength was chosen to provide spectra that are not affected by the plasmonic extinction of Au nanoparticles. However, the Raman spectrum of pristine BDD measured with a 633 nm laser source has been published previously^43^ (the 1% TMBT, 0.2% CO_2_ sample).

### Electrochemical characterization of tested sensors

Cyclic voltammetry (CV) was used for the electrochemical characterization of the sensors. 2.5 mmol L^–1^ K_3_[Fe(CN)_6_] in a 0.1 mol L^–1^ KCl solution served as the first redox marker. Initially, ten cyclic voltammograms were measured using all tested sensors. For BDDE, the potential range was set from the initial potential (*E*_in_) of + 1100 mV to the switching potential (*E*_switch_) of − 500 mV, for SP/BDDE from + 1000 mV to − 700 mV, and for *n*Au-SP/BDDE from + 600 mV to − 500 mV, the scan rate (*v*) was 100 mV s^–1^ for all sensors. The dependences on the *v* were measured in the range of 25–200 mV s^–1^. Another redox system used was a 2.5 mmol L^–1^ [Ru(NH_3_)_6_]Cl_3_ also dissolved in a 0.1 mol L^–1^ KCl solution. The same experiments as for the Fe complex were carried out. Again, the voltammetric parameters varied for different sensors. For the measurement of repeated cyclic voltammograms at *v* of 100 mV s^–1^, the following values were used: *E*_in_ = + 300 mV and *E*_switch_ = − 400 mV for BDDE, *E*_in_ = + 250 mV and *E*_switch_ = − 400 mV for both SP/BDDE and *n*Au-SP/BDDE. Cyclic voltammograms were also measured depending on *v* (25–200 mV s^–1^). The third redox system for testing was DA (50 µmol L^–1^) in BRB solution (pH 5.5). The potential range was set as follows: *E*_in_ = − 1000 mV and *E*_switch_ = + 1500 mV for BDDE, *E*_in_ = − 1000 mV and *E*_switch_ = + 1000 mV for SP/BDDE, *E*_in_ = − 1000 mV and *E*_switch_ = 200 mV for both *n*Au-SP/BDDE and eAu-SP/BDDE. In the case of repeated measurements, a scan rate of 100 mV s^−1^ was used, and the dependence on the *v* was monitored in the range of 25–200 mV s^–1^.

Electrochemical impedance spectroscopy (EIS) experiments were performed in the frequency range from 100 kHz to 0.1 Hz, with a pulse amplitude of 10 mV. Before the EIS experiments, cyclic voltammograms of 2.5 mmol L^−1^ [Fe(CN)_6_]^4−/3−^ in 0.1 mol L^−1^ KCl, 2.5 mmol L^−1^ [Ru(NH_3_)_6_]^2+/3+^ in 0.1 mol L^−1^ KCl and 2.5 mmol L^−1^ DA in 0.1 mol L^−1^ KCl were recorded. The Δ*E*_1/2_ potentials for each sensor type were calculated as half of the difference between the oxidation and reduction peak potentials. The potentials corresponding to Δ*E*_1/2_ values were then applied to the particular electrodes. All EIS measurements were performed 3 times on each type of electrode. The values of particular elements of the equivalent electrical circuits (EECs) R([RW]/Q) for redox markers [Fe(CN)_6_]^4−/3−^ and DA (Fig. S17A), and RW for [Ru(NH_3_)_6_]^2+/3+^ (Fig. S17B) were calculated using FRA simulation in Metrohm NOVA 2.1.5. The fits of the equivalent circuits to the raw data meet the criteria of *χ*^2^ < 0.1.

### Voltammetric analysis of dopamine

Differential pulse voltammetry (DPV) and square wave voltammetry (SWV) were used for the determination of the DA. BRB (pH 5.5) was selected as a suitable supporting electrolyte. Initially, the following parameters were set: *v* = 50 mV s^–1^, pulse height of + 50 mV, and pulse width of 50 ms for DPV; *v* = 50 mV s^–1^, amplitude (*A*) = 50 mV, and frequency (*f*) = 25 Hz for SWV. Due to the higher current response of DA and the higher sensitivity, SWV was chosen for further experiments, and its parameters were optimized (see Optimization of the voltammetric analytical method in Supplementary Material for details) as follows: *v* = 50 mV s^–1^, *A* = 80 mV, and *f* = 10 Hz. The optimized method was used to measure DA concentration dependences on the individual sensors, again differing in the potential ranges used: *E*_in_ = 0 mV and final potential (*E*_fin_) = 1500 mV for BDDE; *E*_in_ = − 500 mV and *E*_fin_ = 700 mV for SP/BDDE; *E*_in_ = − 500 mV and *E*_fin_ = 200 mV for modified electrodes. The resulting statistical parameters were compared. For eAu-SP/BDDE, the measurements were also carried out at a lower *v* (5 mV s^−1^).

DA was determined in model solution and urine sample using unmodified BDDE, SP/BDDE and modified 5Au-SP/BDDE and eAu-SP/BDDE sensors. The concentration of DA in the model solution was 1 µmol L^−1^. The determination was repeated 5 times and the standard addition method was used for evaluation. 10 μL of the DA standard solution with a concentration of 0.001 µmol L^−1^ was added to 10 mL of the sample in the polarographic cell, and 3 standard additions were always made. A urine sample was obtained from a healthy individual not taking any medication. It was then spiked with dopamine to a concentration of 1 µmol L^−1^. Before analysis, the sample was diluted with supporting electrolyte in a 1:1 ratio (5 mL urine, 5 mL BRB (pH 5.5)) and placed in an ultrasonic bath for 15 min^46^. The sample thus prepared was again analyzed by the standard addition method.

### Statistical parameters

Parameters of calibration curves and confidence intervals were calculated at the significance level of 0.05 using QCExpert software (TriloByte, Staré Hradiště, Czech Republic), MS Excel (Microsoft CZ, Prague, Czech Republic), and OriginPro 9.0. The limit of detection (LOD) was calculated from the calibration dependences as three times the standard deviation of an intercept divided by the slope of the calibration line^47,48^.

### Supplementary Information

Supplementary Information.

## Data Availability

Data are available on request from the corresponding author.
