# Supplementary material for: Effect of different modification by gold nanoparticles on the electrochemical performance of screen-printed sensors with boron-doped diamond electrode
Source: Sci Rep. 2023 Dec 6;13:21525. doi: 10.1038/s41598-023-48834-7 (PMC10700314; doi:10.1038/s41598-023-48834-7)
Supplement: Supplementary file 1 — Supplementary Information. [file 41598_2023_48834_MOESM1_ESM.docx]

Supplementary Material

**Effect of different modification by gold nanoparticles on the electrochemical performance of screen-printed sensors with boron-doped diamond electrode**

Oleksandr Matvieiev^1^, Renáta Šelešovská^1,*^, Marián Marton^2^, Michal Hatala^3^, Radovan Metelka^4^, Martin Weis^2^ and Marian Vojs^2^

^1^ University of Pardubice, Faculty of Chemical Technology, Institute of Environmental and Chemical Engineering, Studentská 573, 532 10 Pardubice, Czech Republic

^2^ Slovak University of Technology in Bratislava, Faculty of Electrical Engineering and Information Technology, Institute of Electronics and Photonics, Ilkovičova 3, 812 19 Bratislava, Slovak Republic

^3^ Slovak University of Technology in Bratislava, Faculty of Chemical and Food Technology, Department of Graphic Arts Technology and Applied Photochemistry, Radlinského 9, 812 37 Bratislava, Slovak Republic

^4^ University of Pardubice, Faculty of Chemical Technology, Department of Analytical Chemistry, Studentská 573, 532 10, Pardubice, Czech Republic

^*^ [renata.selesovska@upce.cz](mailto:renata.selesovska@upce.cz)

**
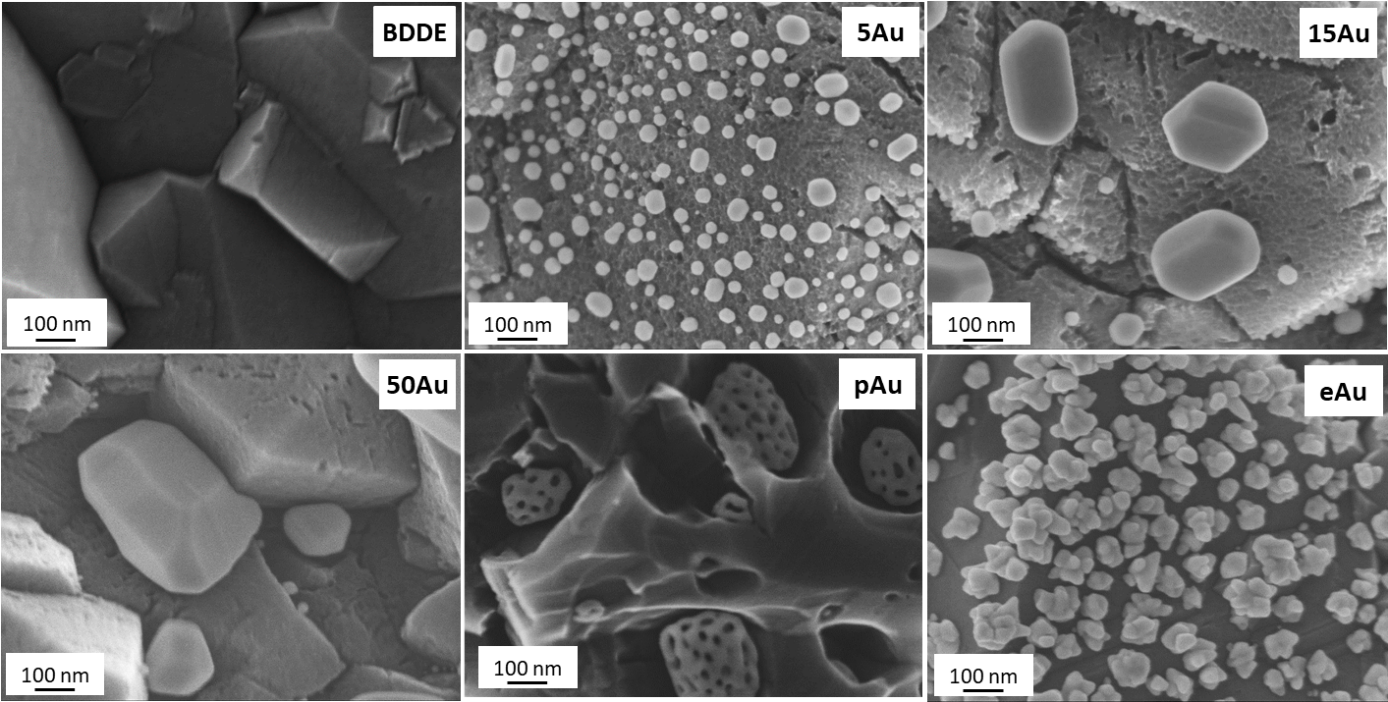
**

**Figure S1** SEM images of unmodified BDD and modified with gold nanoparticles (an increase of 100,000 times)

**
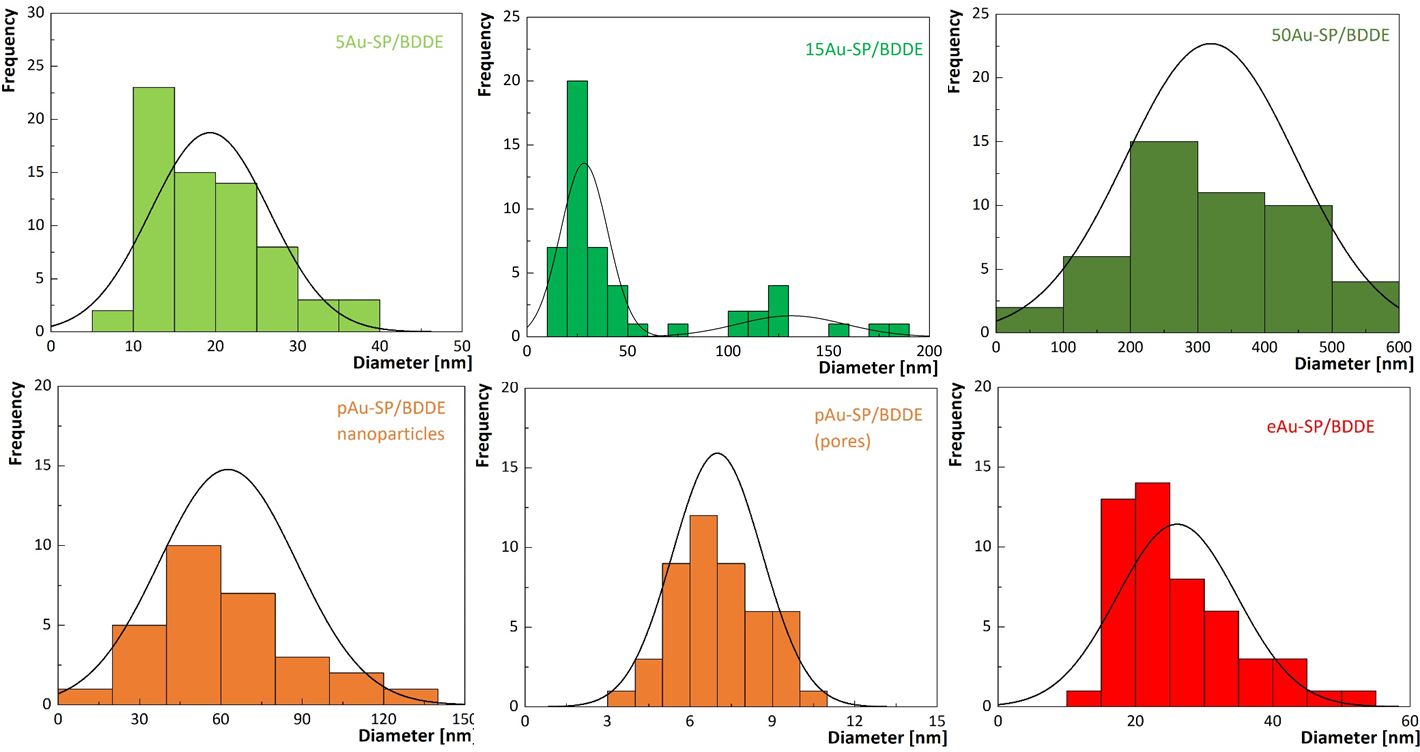
**

**Figure S2** Statistical histograms of the size distribution of gold nanoparticles

**Fabrication of pAuNPs**

The fabrication of pAu nanoparticles on the surface of BDD electrodes consists of three steps (Fig.S3):

1. Au/Ag bilayer is evaporated on the BDD surface using thermal evaporation of several nanometers thin Ag and Au layers in high vacuum in one vacuum cycle.

2. Dewetting of the Ag/Au bilayer using annealing at 600°C for 15 minutes in N_2_ atmosphere to form Au/Ag alloy nanoparticles.

3. Removing Ag from the Au/Ag alloy nanoparticles using wet etching in HF acid to form nanoporous Au nanoparticles.


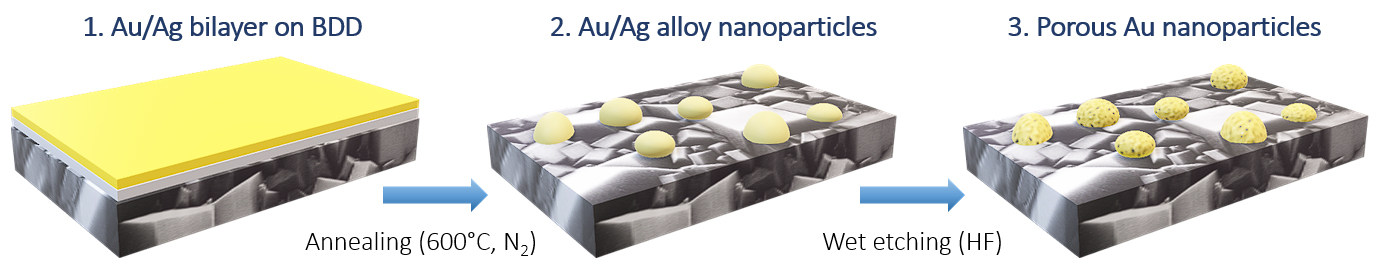


**Figure S3** Simplified representation of the fabrication process of porous Au nanoparticles.

During the fabrication process, the BDD surface was etched to a shallow depth by the Ag/Au alloy nanoparticles (Fig.S3). Even though both gold and silver belong to the noble metal family, the silver is well-known for its easy oxidation ^1^. Therefore, two possible ways of such a thermal catalytic effect are considered possible. First, it is highly likely, that oxygen is adsorbed at the surface of the Ag component of the Au/Ag bilayer between the vacuum evaporation and annealing steps of the fabrication process when it is exposed to air. Therefore, when the BDD/Au/Ag structure is annealed to form the Au/Ag alloy nanoparticles, the oxygen is released from Ag, promoting a thermal etching process of the BDD surface, similar to that observed in ^2^. Second, a thermally assisted catalytic etching of the BDD surface by forming silver carbide (Ag_2_C_2_) is considered possible as well.

**Table S1** Values of I(G)/I(ZCP_D_) calculated from Raman spectra measured with 325 nm laser source wavelength.

| Electrode | I (G) / I (ZCP_D_) |
| --- | --- |
| BDDE | 0.28 |
| 5Au-SP/BDDE | 0.27 |
| 15Au-SP/BDDE | 0.31 |
| 50Au-SP/BDDE | 0.28 |
| eAu-SP/BDDE | 0.30 |
| pAu-SP/BDDE | 0.29 |


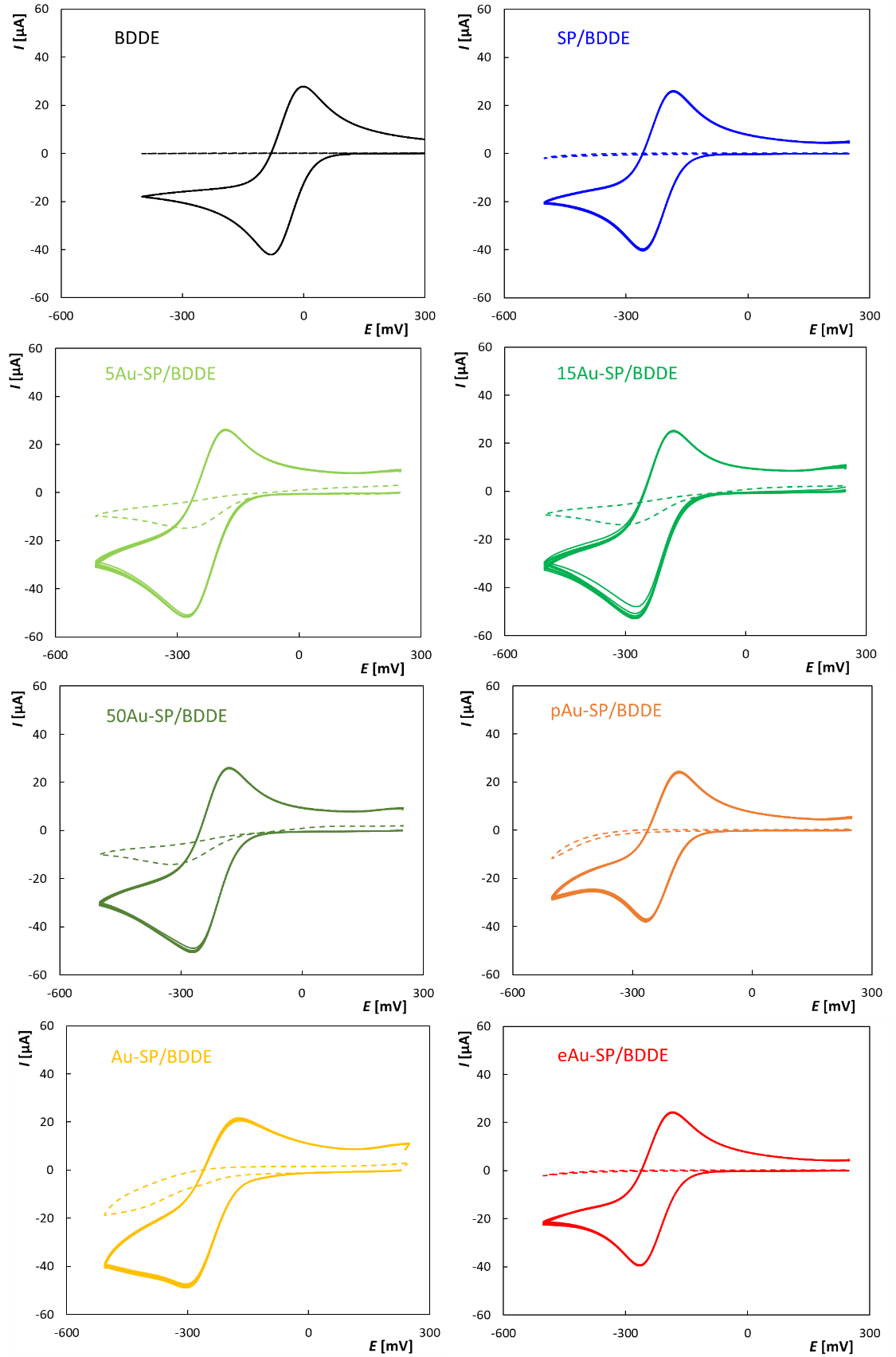


**Figure S4** Cyclic voltammograms of Ru(NH_3_)_6_]^2+/3+^ recorded on all tested sensors (electrolyte – 0.1 mol L^−1^ KCl, *v* = 100 mV s^−1^, *c*(Ru(NH_3_)_6_]^2+/3+^) = 2.5 mmol L^−1^).


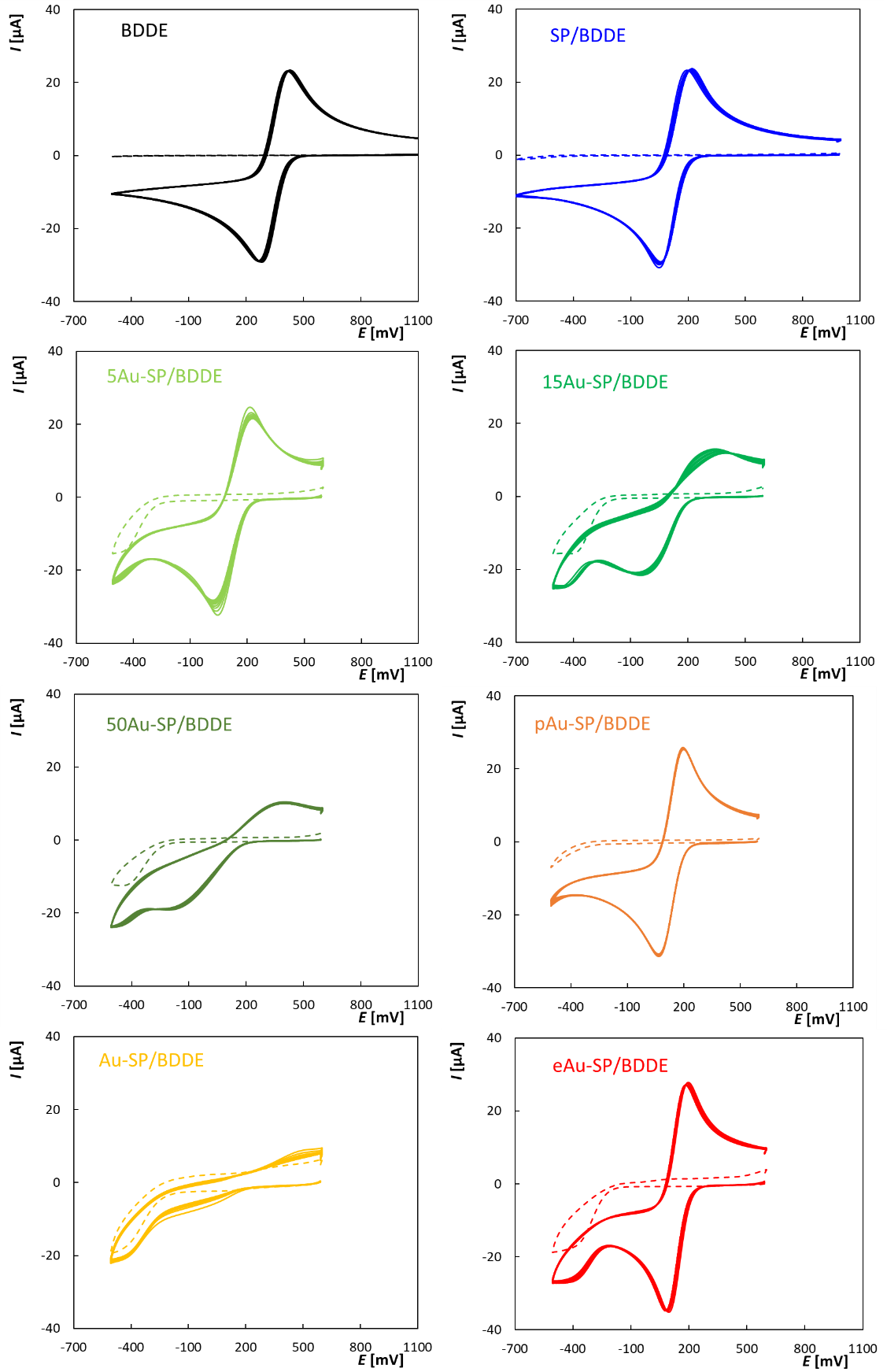


**Figure S5** Cyclic voltammograms of [Fe(CN)_6_]^4−/3−^ recorded on all tested sensors (electrolyte – 0.1 mol L^−1^ KCl, *v* = 100 mV s^−1^, *c*([Fe(CN)_6_]^4−/3−^) = 2.5 mmol L^−1^).


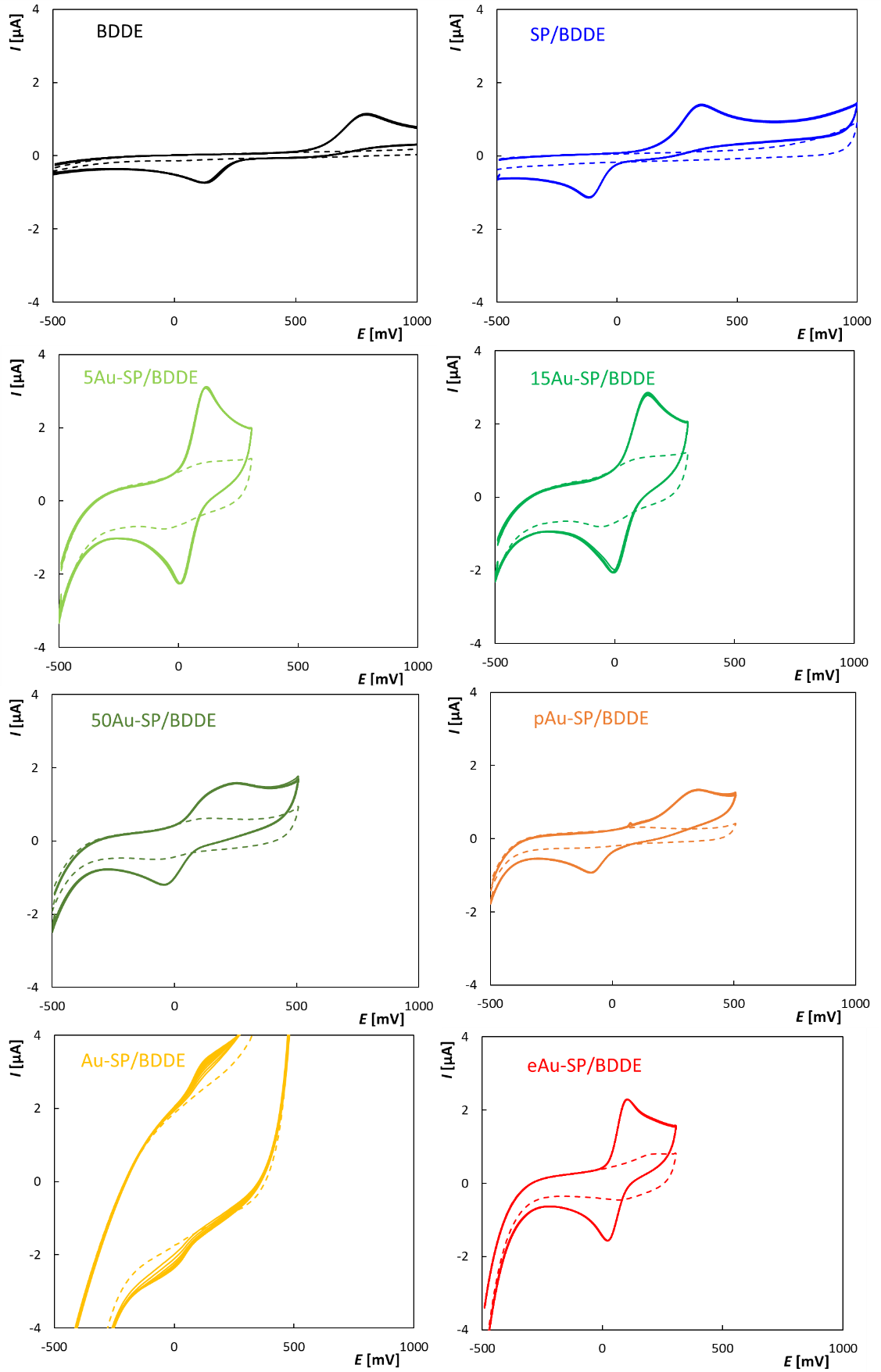


**Figure S6** Cyclic voltammograms of DA recorded on all tested sensors (electrolyte – BRB (pH 5.5), *v* = 100 mV s^−1^, *c*(DA) = 50 µmol L^−1^).

**Table S2** Parameters of cyclic voltammograms of redox marker [Ru(NH_3_)_6_]^2+/3+^, Fe(CN)_6_]^4−/3−^, and DA (electrolyte – 0.1 mol L^−1^ KCl and BRB (pH 5.5), *v* = 25-200 mV s^−1^, *c*([Fe(CN)_6_]^4−/3−^) = 2.5 mmol L^−1^, *c*([Ru(NH_3_)_6_]^2+/3+^) = 2.5 mmol L^−1^, *c*(DA) = 50 µmol L^−1^).

| **Electrode** | ***I*_pa_**  **[µA]** | ***I*_pc_**  **[µA]** | ***I*_pa_/*I*_pc_** | ***E*_pa_**  **[mV]** | ***E*_pc_**  **[mV]** | **Δ*E*_p_**  **[mV]** |
| --- | --- | --- | --- | --- | --- | --- |
| [Ru(NH_3_)_6_]^2+/3+^ |  |  |  |  |  |  |
| BDDE | 34.6 | –35.7 | 1.0 | –5.49 | –78.89 | 73.4 |
| SP/BDDE | 35.2 | –31.9 | 1.1 | –184.48 | –255.89 | 71.4 |
| 5Au-SP/BDDE | 33.0 | –37.0 | 0.9 | –188.45 | –269.78 | 81.3 |
| 15Au-SP/BDDE | 32.4 | –37.5 | 0.9 | –184.48 | –265.81 | 81.3 |
| 50Au-SP/BDDE | 33.2 | –36.6 | 0.9 | –184.48 | –263.82 | 79.3 |
| pAu-SP/BDDE | 33.3 | –26.7 | 1.3 | –186.46 | –259.86 | 73.4 |
| Au-SP/BDDE | 29.8 | -27.6 | 1.1 | –183.11 | –293.88 | 110.8 |
| eAu-SP/BDDE | 33.5 | –30.1 | 1.1 | –186.46 | –259.89 | 73.4 |
| [Fe(CN)_6_]^4−/3−^ |  |  |  |  |  |  |
| BDDE | 26.9 | –26.1 | 1.0 | 404.4 | 261.8 | 142.6 |
| SP/BDDE | 27.6 | –26.1 | 1.1 | 214.5 | 53.4 | 161.1 |
| 5Au-SP/BDDE | 21.5 | –20.5 | 1.1 | 227.4 | 36.0 | 191.3 |
| 15Au-SP/BDDE | 12.6 | –14.1 | 0.9 | 257.6 | 15.9 | 241.7 |
| 50Au-SP/BDDE | 6.4 | –5.0 | 1.3 | 338.1 | –115.1 | 453.2 |
| pAu-SP/BDDE | 27.8 | –26.1 | 1.1 | 197.1 | 66.2 | 130.9 |
| Au-SP/BDDE | 0.552 | –0.327 | 1.7 | 177.0 | 86.4 | 90.6 |
| eAu-SP/BDDE | 29.4 | –28.3 | 1.0 | 177.0 | 86.4 | 90.6 |
| DA |  |  |  |  |  |  |
| BDDE | 0.797 | −0.568 | 1.4 | 779.0 | 114.3 | 664.7 |
| SP/BDDE | 0.939 | −0.827 | 1.1 | 345.9 | −117.3 | 463.3 |
| 5Au-SP/BDDE | 1.755 | −2.021 | 0.9 | 114.3 | 3.5 | 110.8 |
| 15Au-SP/BDDE | 1.393 | −1.783 | 0.8 | 134.4 | −6.6 | 141.0 |
| 50Au-SP/BDDE | 0.597 | −0.824 | 0.7 | 215.0 | −36.8 | 251.8 |
| pAu-SP/BDDE | 0.478 | −0.593 | 0.8 | 335.9 | −87.1 | 423.0 |
| Au-SP/BDDE | 0.378 | −0.238 | 1.6 | 124.4 | −6.6 | 130.9 |
| eAu-SP/BDDE | 1.425 | −1.428 | 1.0 | 104.2 | 23.7 | 80.6 |


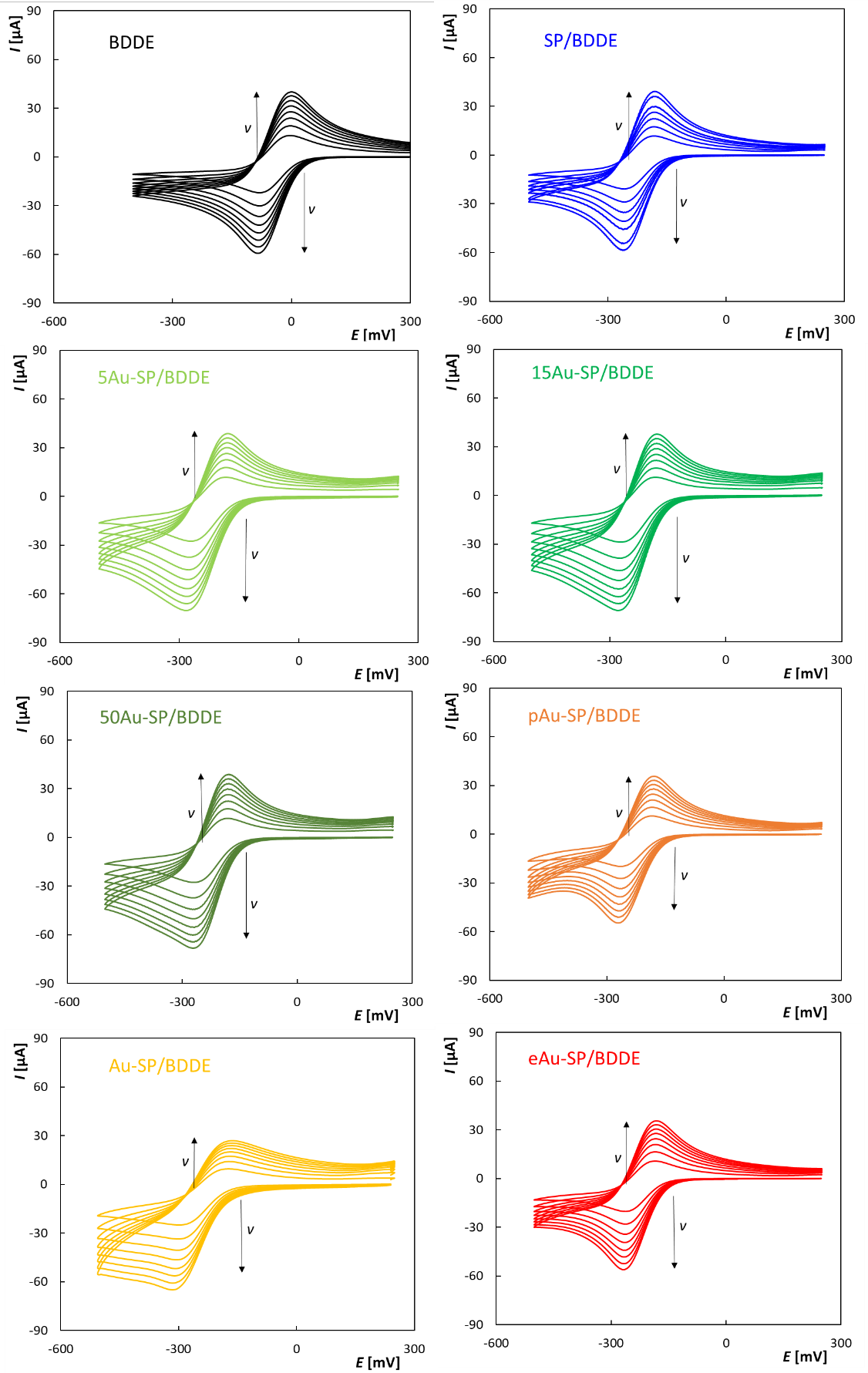


**Figure S7** Cyclic voltammograms of [Ru(NH_3_)_6_]^2+/3+^ recorded on tested sensors at various scan rates (electrolyte – 0.1 mol L^−1^ KCl, *v* = 25-200 mV s^−1^, *c*([Ru(NH_3_)_6_]^2+/3+^) = 2.5 mmol L^−1^).

*
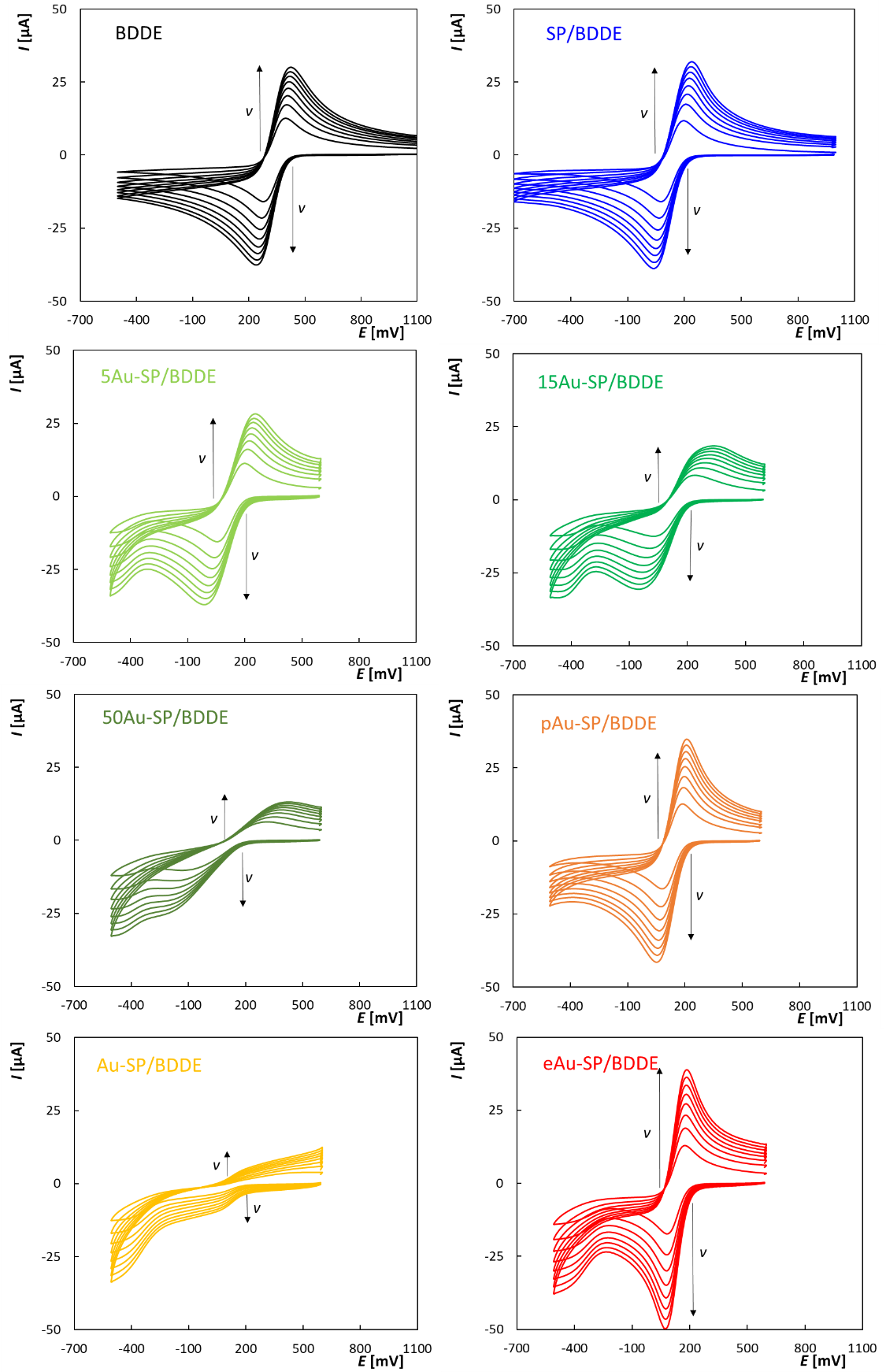
*

**Figure S8** Cyclic voltammograms of [Fe(CN)_6_]^4−/3−^ recorded on tested sensors at various scan rates (electrolyte – 0.1 mol L^−1^ KCl, *v* = 25-200 mV s^−1^, *c*([Fe(CN)_6_]^4−/3−^) =2.5 mmol L^−1^).


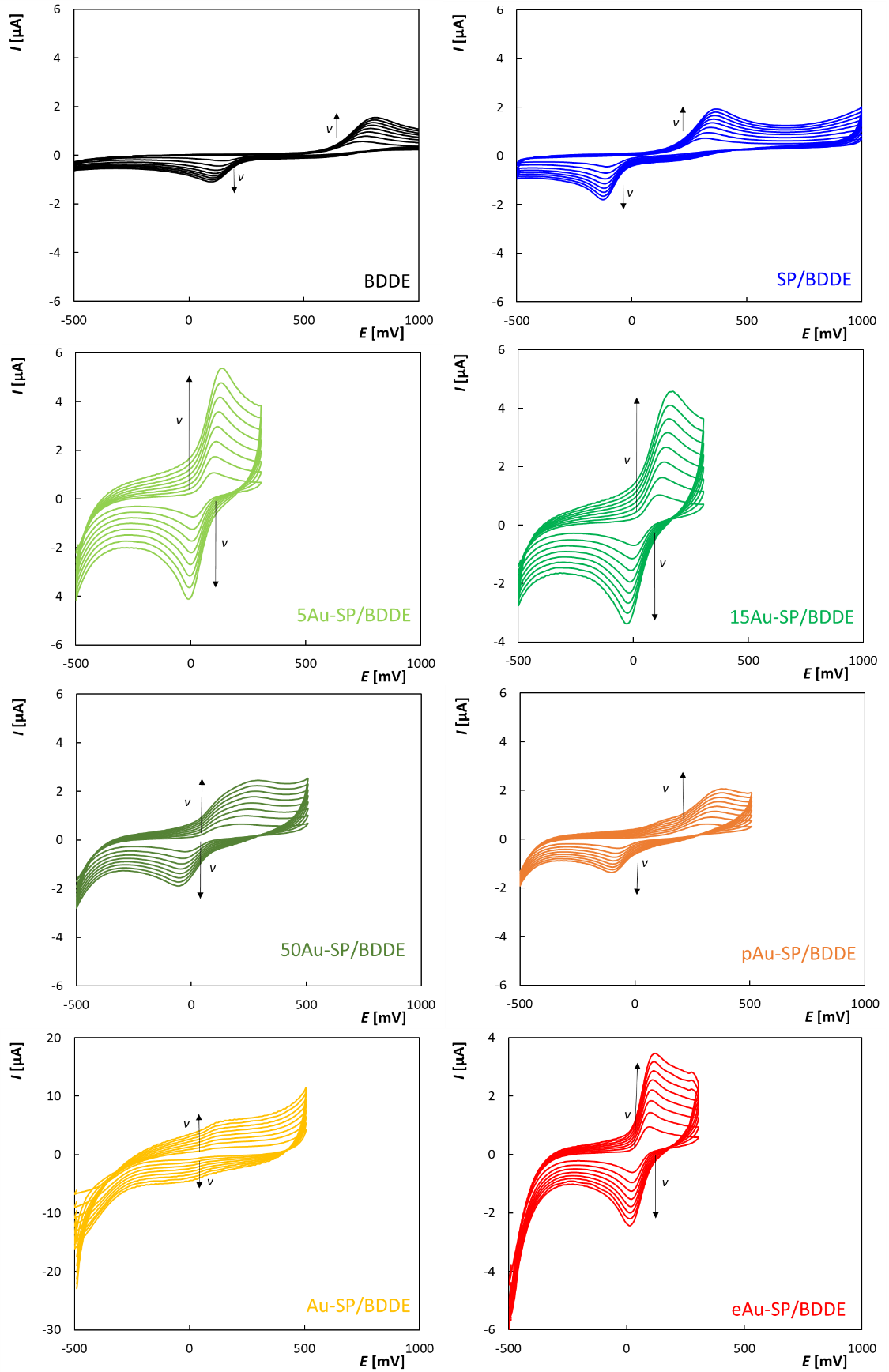


**Figure S9** Cyclic voltammograms of DA recorded on tested sensors at various scan rates (electrolyte – BRB (pH 5.5), *v* = 25-200 mV s^−1^, *c*(DA)= 50 µmol L^−1^).

**Table S3** Statistical parameters of dependences of *I*_p_ on *v*^1/2^ for[Ru(NH_3_)_6_]^2+/3+^, [Fe(CN)_6_]^4−/3−^, and DA (electrolyte – 0.1 mol L^−1^ KCl and BRB (pH 5.5), *v* = 25-200 mV s^−1^, *c*([Fe(CN)_6_]^4−/3−^) = 2.5 mmol L^−1^, *c*([Ru(NH_3_)_6_]^2+/3+^) = 2.5 mmol L^−1^, *c*(DA) = 50 µmol L^−1^).

| **Electrode** | **Slope (a)** | **Intercept (a)** | ***r*** | **Slope (c)** | **Intercept (c)** | ***r*** |
| --- | --- | --- | --- | --- | --- | --- |
|  | **[µA s^1/2^ mV^−1/2^]** | **[µA]** |  | **[µA s^1/2^ mV^−1/2^]** | **[µA]** |  |
| [Ru(NH_3_)_6_]^2+/3+^ |  |  |  |  |  |  |
| BDDE | (3.36 ± 0.012) | (0.925 ± 0.134) | 0.9999 | −(3.54 ± 0.015) | −(0.314 ± 0.153) | 0.9999 |
| SP/BDDE | (3.75 ± 0.051) | −(2.08 ± 0.536) | 0.9995 | −(3.33 ± 0.032) | −(1.13 ± 0.344) | 0.9997 |
| 5Au-SP/BDDE | (3.98 ± 0.023) | −(2.28 ± 0.241) | 0.9999 | −(3.16 ± 0.037) | −(5.10 ± 0.393) | 0.9996 |
| 15Au-SP/BDDE | (4.08 ± 0.067) | −(2.99 ± 0.393) | 0.9992 | −(3.11 ± 0.046) | −(6.21 ± 0.493) | 0.9993 |
| 50Au-SP/BDDE | (3.84 ± 0.187) | −(0.481 ± 1.984) | 0.9940 | −(3.09 ± 0.030) | −(5.44 ± 0.317) | 0.9997 |
| pAu-SP/BDDE | (3.44 ± 0.031) | −(0.766 ± 0.327) | 0.9998 | −(2.79 ± 0.031) | (1.70 ± 0.327) | 0.9996 |
| Au-SP/BDDE | (2.46 ± 0.078) | (4.46 ± 0.826) | 0.9970 | −(2.21 ± 0.029) | −(5.22 ± 0.312) | 0.9995 |
| eAu-SP/BDDE | (3.32 ± 0.014) | (0.165 ± 0.154) | 0.9999 | −(3.09 ± 0.024) | (0.675 ± 0.251) | 0.9998 |
| [Fe(CN)_6_]^4−/3−^ |  |  |  |  |  |  |
| BDDE | (2.17 ± 0.048) | (4.92 ± 0.511) | 0.9985 | −(2.10± 0.054) | −(4.81 ± 0.537) | 0.9981 |
| SP/BDDE | (2.34 ± 0.037) | (3.93 ± 0.390) | 0.9993 | −(2.18 ± 0.041) | −(4.03 ± 0.436) | 0.9990 |
| 5Au-SP/BDDE | (1.49 ± 0.056) | (6.24 ± 0.591) | 0.9958 | −(1.39 ± 0.057) | −(6.13 ± 0.604) | 0.9950 |
| 15Au-SP/BDDE | (0.642 ± 0.016) | (6.03 ± 0.168) | 0.9982 | −(0.981 ± 0.019) | −(4.16 ± 0.199) | 0.9989 |
| 50Au-SP/BDDE | (0.110 ± 0.019) | (5.25 ± 0.197) | 0.9241 | −(0.072 ± 0.018) | −(4.24 ± 0.188) | 0.8557 |
| pAu-SP/BDDE | (2.46 ± 0.047) | (2.94 ± 0.495) | 0.9989 | −(2.15 ± 0.062) | −(4.12 ± 0.652) | 0.9976 |
| Au-SP/BDDE | (0.063 ± 0.004) | (0.046 ± 0.037) | 0.9906 | −(0.063 ± 0.037) | −(0.058 ± 0.007) | 0.9521 |
| eAu-SP/BDDE | (2.86 ± 0.016) | (0.731 ± 0.171) | 0.9999 | −(2.87 ± 0.006) | (0.352 ± 0.066) | 0.9999 |
| Dopamine |  |  |  |  |  |  |
| BDDE | (3.36 ± 0.013) | (0.925 ± 0.134) | 0.9999 | −(3.54 ± 0.014) | –(0.314 ± 0.15) | 0.9999 |
| SP/BDDE | (3.75 ± 0.051) | –(2.08 ± 0.537) | 0.9995 | −(3.33 ± 0.032) | (1.13± 0.344) | 0.9997 |
| 5Au-SP/BDDE | (3.98 ± 0.023) | −(2.28 ± 0.241) | 0.9999 | −(3.16 ± 0.037) | –(5.10 ± 0.393) | 0.9996 |
| 15Au-SP/BDDE | (4.08 ± 0.067) | −(2.99 ± 0.713) | 0.9992 | −(3.11 ± 0.046) | –(6.21 ± 0.493) | 0.9993 |
| 50Au-SP/BDDE | (3.85 ± 0.187) | −(0.481 ± 1.984) | 0.9930 | −(3.09± 0.030) | –(5.44 ± 0.317) | 0.9997 |
| pAu-SP/BDDE | (3.44 ± 0.031) | −(0.766 ± 0.327) | 0.9998 | −(2.79 ± 0.032) | (1.70 ± 0.334) | 0.9996 |
| Au-SP/BDDE | (0.066 ± 0.004) | −(0.065 ± 0.045) | 0.9872 | −(0.059 ± 0.008) | – (0.219 ± 0.084) | 0.9502 |
| eAu-SP/BDDE | (3.32 ± 0.015) | (0.165 ± 0.154) | 0.9999 | −(3.09 ± 0.024) | (0.675 ± 0.251) | 0.9998 |

a – anodic peak, c – cathodic peak

**Table S4** Statistical parameters of dependences of log(*I*_p_) on log(*v*) for[Ru(NH_3_)_6_]^2+/3+^, [Fe(CN)_6_]^4−/3−^, and DA (electrolyte – 0.1 mol L^−1^ KCl and BRB (pH 5.5), *v* = 25-200 mV s^−1^, *c*([Fe(CN)_6_]^4−/3−^) = 2.5 mmol L^−1^, *c*([Ru(NH_3_)_6_]^2+/3+^) = 2.5 mmol L^−1^, *c*(DA) = 50 µmol L^−1^).

| **Electrode** | **Slope (a)** | ***r*** | **Slope (c)** | ***r*** |
| --- | --- | --- | --- | --- |
| [Ru(NH_3_)_6_]^2+/3+^ |  |  |  |  |
| BDDE | (0.486 ± 0.001) | 0.9999 | (0.492 ± 0.003) | 0.9999 |
| SP/BDDE | (0.529 ± 0.005) | 0.9997 | (0.215 ± 0.004) | 0.9998 |
| 5Au-SP/BDDE | (0.534 ± 0.001) | 0.9999 | (0.424 ± 0.001) | 0.9999 |
| 15Au-SP/BDDE | (0.543 ± 0.006) | 0.9996 | (0.410 ± 0.002) | 0.9999 |
| 50Au-SP/BDDE | (0.520 ± 0.018) | 0.9963 | (0.416 ± 0.002) | 0.9999 |
| pAu-SP/BDDE | (0.515 ± 0.004) | 0.9998 | (0.534 ± 0.005) | 0.9997 |
| Au-SP/BDDE | (0.423 ± 0.008) | 0.9989 | (0.394 ± 0.003) | 0.9998 |
| eAu-SP/BDDE | (0.500 ± 0.002) | 0.9999 | (0.508 ± 0.003) | 0.9999 |
| [Fe(CN)_6_]^4−/3−^ |  |  |  |  |
| BDDE | (0.402 ± 0.004) | 0.9997 | (0.403 ± 0.005) | 0.9995 |
| SP/BDDE | (0.423 ± 0.003) | 0.9999 | (0.418 ± 0.004) | 0.9998 |
| 5Au-SP/BDDE | (0.346 ± 0.006) | 0.9991 | (0.342 ± 0.007) | 0.9986 |
| 15Au-SP/BDDE | (0.241 ± 0.002) | 0.9987 | (0.339 ± 0.002) | 0.9999 |
| 50Au-SP/BDDE | (0.084 ± 0.009) | 0.9652 | (0.071 ± 0.013) | 0.9112 |
| pAu-SP/BDDE | (0.445 ± 0.005) | 0.9996 | (0.419 ± 0.007) | 0.9991 |
| Au-SP/BDDE | (0.543 ± 0.024) | 0.9941 | (0.730 ± 0.072) | 0.9723 |
| eAu-SP/BDDE | (0.486 ± 0.002) | 0.9999 | (0.507 ± 0.001) | 0.9999 |
| Dopamine |  |  |  |  |
| BDDE | (0.486 ± 0.001) | 0.9999 | (0.492 ± 0.006) | 0.9999 |
| SP/BDDE | (0.529 ± 0.005) | 0.9997 | (0.515 ± 0.004) | 0.9998 |
| 5Au-SP/BDDE | (0.534 ± 0.001) | 0.9999 | (0.424 ± 0.001) | 0.9999 |
| 15Au-SP/BDDE | (0.543 ± 0.006) | 0.9996 | (0.543 ± 0.006) | 0.9999 |
| 50Au-SP/BDDE | (0.520 ± 0.018) | 0.9963 | (0.416 ± 0.002) | 0.9999 |
| pAu-SP/BDDE | (0.515 ± 0.004) | 0.9998 | (0.534 ± 0.005) | 0.9997 |
| Au-SP/BDDE | (0.543 ± 0.025) | 0.9939 | (0.740 ± 0.074) | 0.9712 |
| eAu-SP/BDDE | (0.499 ± 0.002) | 0.9999 | (0.508 ± 0.003) | 0.9998 |

a – anodic peak, c – cathodic peak


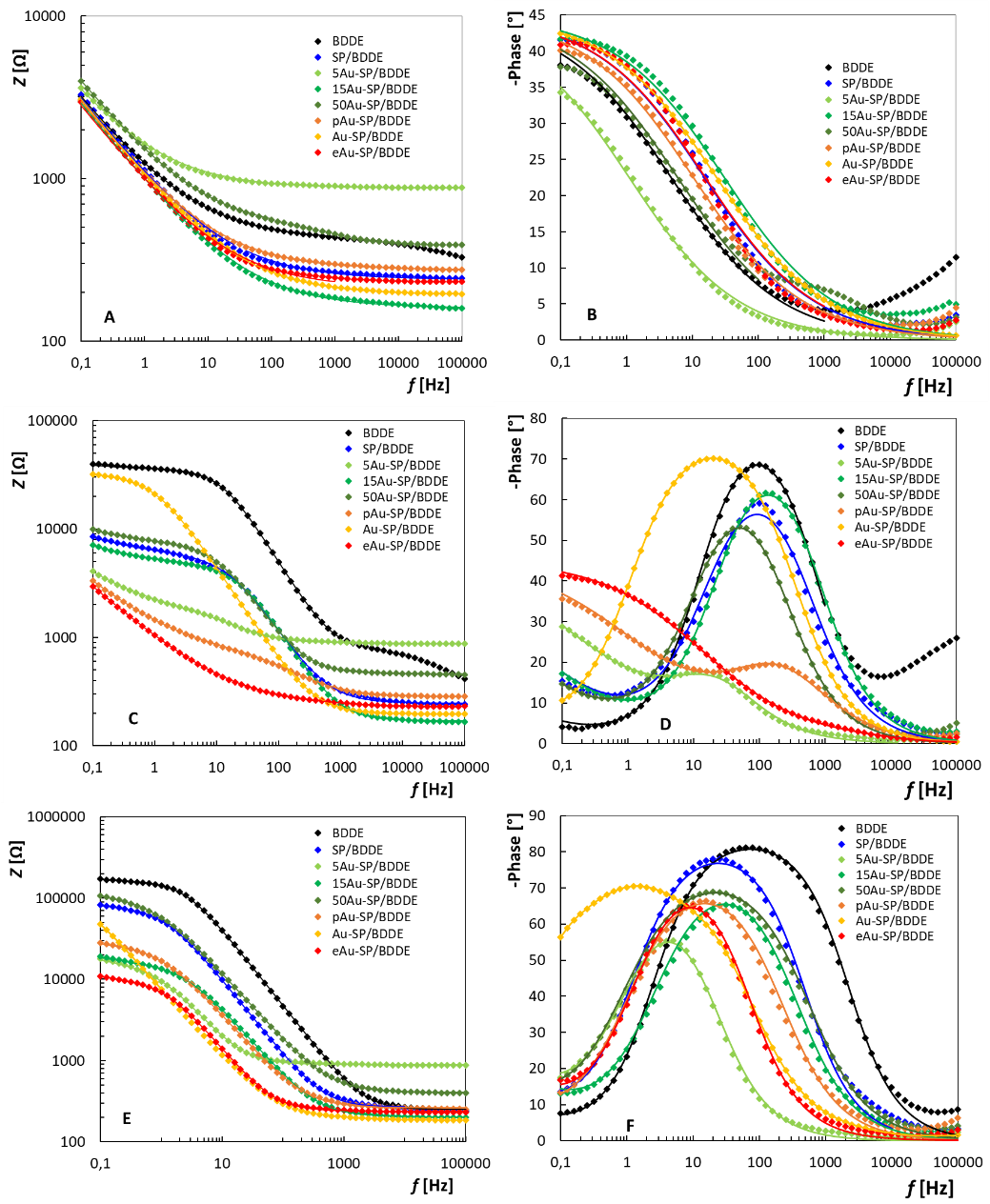


**Figure S10** The Bode impedance plots (A, C, E) and the Bode phase plots (B, D, F) of the data for each electrode resulting from an EIS measurement performed at 2.5 mmol L^−1^ [Ru(NH_3_)_6_]^2+/3+^ (A, B), 2.5 mmol L^−1^ [Fe(CN)_6_]^4−/3−^ (C, D) 2.5 mmol L^−1^ DA (E, F) in 0.1 mol L^−1^ KCl (*f* = 0.1-100k Hz, *A* = 10 mV).

**Table S5** The values of the particular elements of the *RW* for the [Ru(NH_3_)_6_]^2+/3+^ redox marker calculated using the FRA simulation software (2.5 mmol L^−1^ [Ru(NH_3_)_6_]^2+/3+^, in 0.1 M KCl, *f* = 0.1-100k Hz, *A* = 10 mV).

| **Electrode** | ***R*_s_**  **[Ω]** | ***Y*_0_**  **[µMh s^1/2^]** | ***σ*_W_**  **[Ω m^2^/s^1/2^]** | ***k*^0^_app_ [10^6^cm/s]** | ***χ*^2^** |
| --- | --- | --- | --- | --- | --- |
| BDDE | 410.8 | 435 | 103.7 | 3.66 | 0.01 |
| SP/BDDE | 182.0 | 407 | 112.9 | 8.27 | 0.01 |
| 5Au-SP/BDDE | 808.4 | 439 | 108.3 | 1.86 | 0.02 |
| 15Au-SP/BDDE | 123.3 | 451 | 105.2 | 12.2 | 0.07 |
| 50Au-SP/BDDE | 463.9 | 344 | 134.2 | 3.25 | 0.01 |
| pAu-SP/BDDE | 242.2 | 453 | 103.7 | 6.21 | 0.03 |
| Au-SP/BDDE | 182.5 | 428 | 107.8 | 8.24 | 0.01 |
| eAu-SP/BDDE | 147.14 | 473 | 103.7 | 10.2 | 0.08 |

Abbreviations: *R*_s_ – serial resistance, *Y*_0_ – admittance, σ_W_ – Warburg coefficient, *k*^0^_app_ – apparent heterogeneous electron-transfer rate constants, χ^2^ – criterion for assessing the correctness of fitting and simulation.

**Table S6** The values of the particular elements of the *R*(*Q*/[*RW*]) for the [Fe(CN)_6_]^4−/3−^ and DA redox markers calculated using the FRA simulation software (2.5 mmol L^−1^, [Fe(CN)_6_]^4−/3−^ and Dopamine in 0.1 M KCl, *f* = 0.1-100k Hz, *A* = 10 mV).

| **Electrode** | ***R*_s_**  **[Ω]** | ***R*_ct_**  **[kΩ]** | ***Y*_0_**  **[µMh s^1/2^]** | ***Y*_0_**  **[µMh s^n^]** | ***n*** | ***C*_eff_ [µF/cm^2^]** | ***k*^0^_app_ [10^6^cm/s]** | ***τ***  **[ms]** | **χ^2^** |
| --- | --- | --- | --- | --- | --- | --- | --- | --- | --- |
| **[Fe(CN)_6_]^4−/3−^** |  |  |  |  |  |  |  |  |  |
| BDDE | 762 | 34.8 | 266 | 474 | 0.942 | 32.7 | 0.04 | 80.4 | 0.01 |
| SP/BDDE | 247 | 55.2 | 360 | 3.37 | 0.870 | 4.2 | 0.03 | 16.4 | 0.09 |
| 5Au-SP/BDDE | 926 | 0.9 | 458 | 19.4 | 0.891 | 9.3 | 1.67 | 0.59 | 0.01 |
| 15Au-SP/BDDE | 169 | 4.6 | 427 | 2.5 | 0.907 | 2.7 | 0.33 | 0.88 | 0.01 |
| 50Au-SP/BDDE | 461 | 6.9 | 378 | 3.57 | 0.883 | 4.2 | 0.22 | 2.04 | 0.03 |
| pAu-SP/BDDE | 283 | 0.5 | 440 | 17.8 | 0.737 | 26.8 | 3.16 | 0.94 | 0.01 |
| Au-SP/BDDE | 196 | 31.2 | 431 | 5.97 | 0.877 | 5.1 | 0.09 | 11.2 | 0.01 |
| eAu-SP/BDDE | 229 | 0.02 | 444 | 12.7 | 0.820 | 6.65 | 80.9 | 0.01 | 0.01 |
| **DA** |  |  |  |  |  |  |  |  |  |
| BDDE | 250 | 157 | 68,6 | 472 | 0.947 | 29.6 | 0.01 | 328 | 0.09 |
| SP/BDDE | 270 | 71.9 | 80 | 2.14 | 0.929 | 2.2 | 0.02 | 11.2 | 0.09 |
| 5Au-SP/BDDE | 898 | 14.1 | 260 | 14.8 | 0.869 | 10.6 | 0.11 | 10.5 | 0.04 |
| 15Au-SP/BDDE | 204 | 15.1 | 255 | 6.05 | 0.866 | 5.6 | 0.10 | 5.98 | 0.05 |
| 50Au-SP/BDDE | 406 | 97.7 | 60.8 | 2.58 | 0.839 | 5.1 | 0.01 | 35.2 | 0.08 |
| pAu-SP/BDDE | 262 | 25.8 | 345 | 8.09 | 0.839 | 8.2 | 0.06 | 14.9 | 0.09 |
| Au-SP/BDDE | 706 | 12.7 | 7.15 | 27.2 | 0.861 | 14.9 | 0.12 | 13.4 | 0.01 |
| eAu-SP/BDDE | 238 | 9.19 | 490 | 17.0 | 0.910 | 7.1 | 0.16 | 4.61 | 0.05 |

Abbreviations: *R*_s_ – serial resistance, *R*_ct_ – charge transfer resistance, *Y*_0_ –admittance, *n* – exponent of constant phase element, *C*_eff_ – double layer capacitance*, k*^0^_app_ – apparent heterogeneous electron-transfer rate constants, τ – relaxation time, χ^2^ – criterion for assessing the correctness of fitting and simulation.


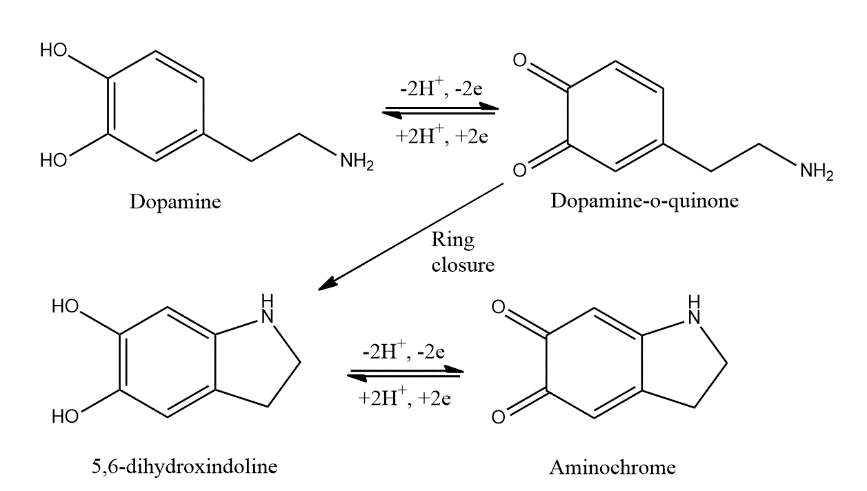


**Figure S11** Mechanism of electrochemical oxidation of dopamine ^3^

**Optimization of the voltammetric analytical method**

The voltammetric method for the determination of DA was optimized using BDDE. The composition of the supporting electrolyte, and especially the pH, significantly affects the electrochemical behavior of investigated substances. Figure S12 shows the cyclic voltammograms of DA obtained in Britton-Robinson buffer (BRB) as an electrolyte within the pH range of 3-8. It is clear that the best-developed peak of DA oxidation was observed in a weakly acidic environment (3-6). Therefore, BRB (pH 5.5) was chosen for the following measurements. Further experiments confirmed that this environment is also suitable for working with AuNPs modified electrodes. Differential pulse voltammetry (DPV) and square wave voltammetry (SWV) were initially tested for DA determination. To compare these methods, the concentration dependences of DA were measured using BDDE in the range of 2-10 μmol L^–1^ (Figure S13). It is obvious that SWV provides significantly higher current responses and the value of calibration slope 18.53±0.11 is twice as high as for DPV 9.739±0.078 nA L µmol^−1^. Therefore, the SWV with the following optimized parameters, such as *v* = 50 mV s^–1^, *A* = 80 mV, and *f* = 10 Hz, was used to determine DA using all tested sensors.


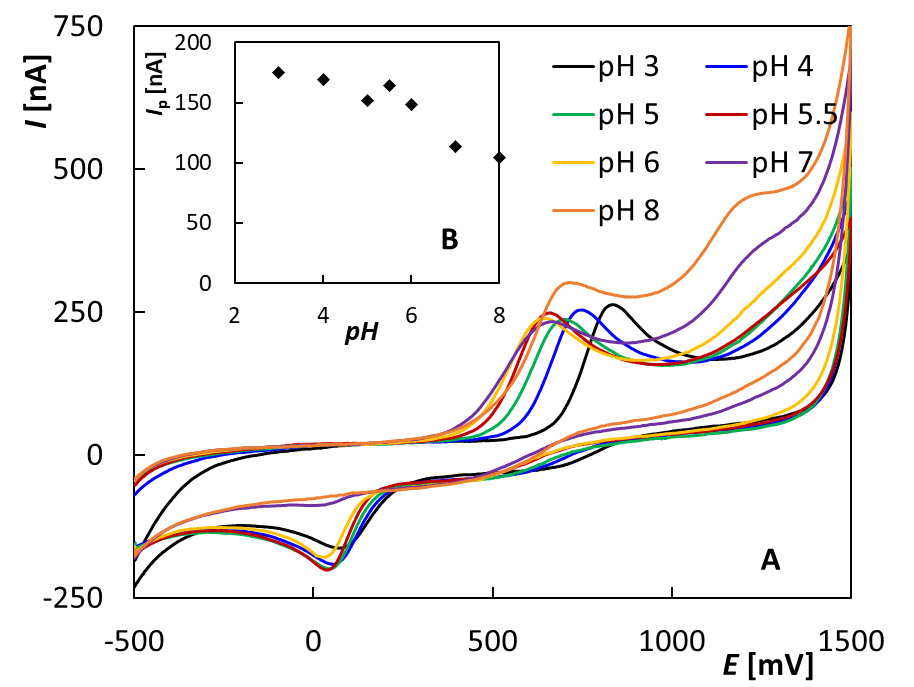


**Figure S12** Cyclic voltammograms of DA recorded on BDDE as a function of pH (A) and dependence of the height of the oxidation peak on pH (B); electrolyte BRB (pH 3-8), c(DA) = 10 μmol L^–1^, *ν* = 100 mV s^–1^.


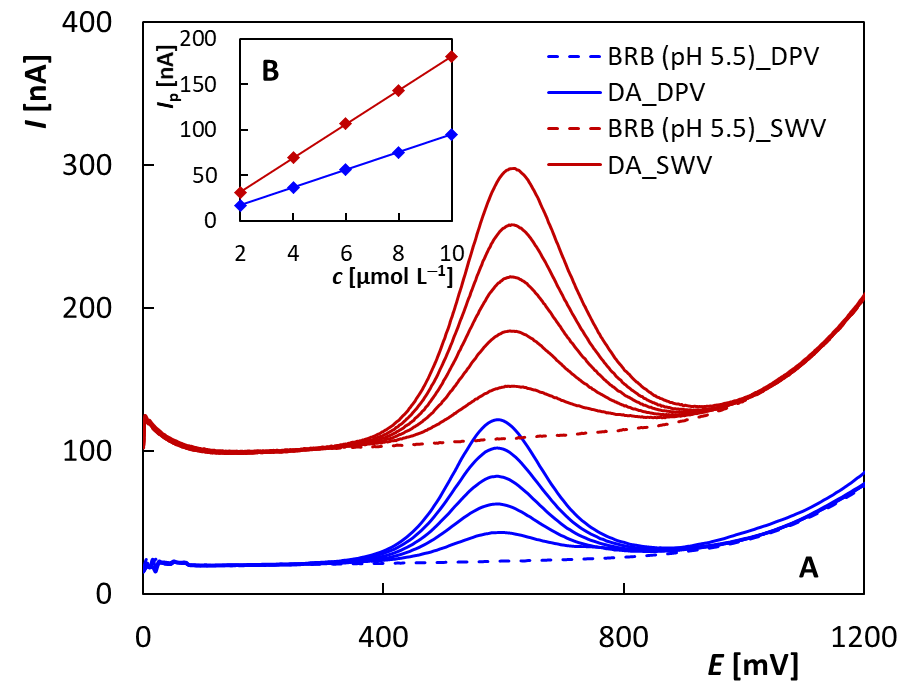


**Figure S13** Comparison of DP and SW voltammetric curves of DA recorded on BDDE as a function of concentration (A) and the corresponding dependence of *I*_p_ on c(DA); electrolyte – BRB (pH 5.5), *c*(DA) = 2-10 μmol L^–1^, DPV: *ν* = 50 mV s^–1^, pulse height = +50 mV, pulse duration = 50 ms; SWV: *v* = 50 mV s^–1^, *A* = 50 mV, *f* = 25 Hz.


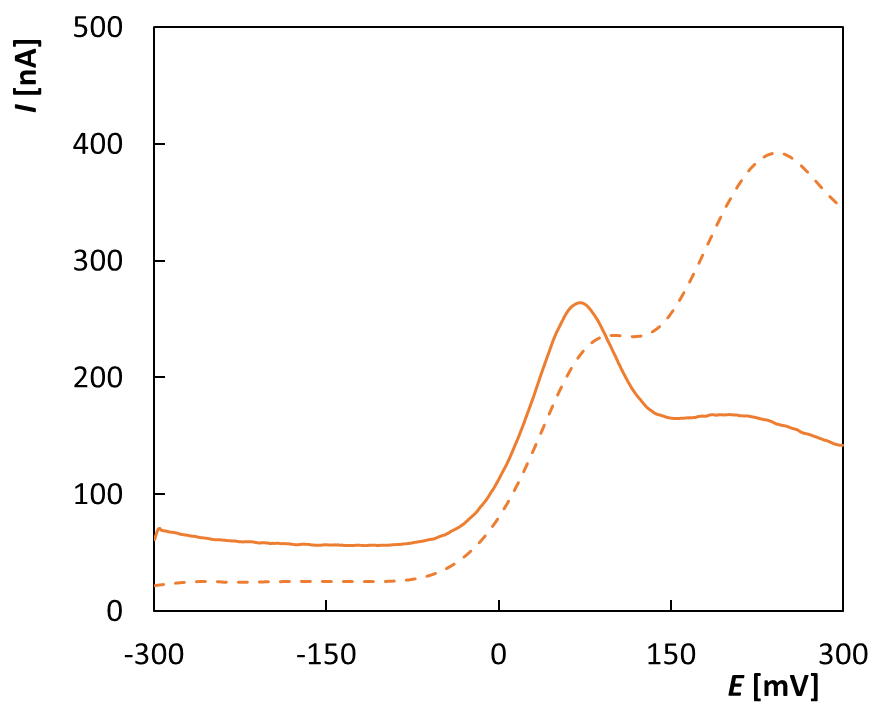


**Figure S14** SW voltammograms of DA recorded on a pAu-SP/BDDE; electrolyte – BRB (pH 5.5), ν = 50 mV s^–1^ (dash line) and 5 mV s^–1^ (solid line), *A* = 80 mV, *f* = 10 Hz, *c*(DA) = 15 μmol L^–1^.


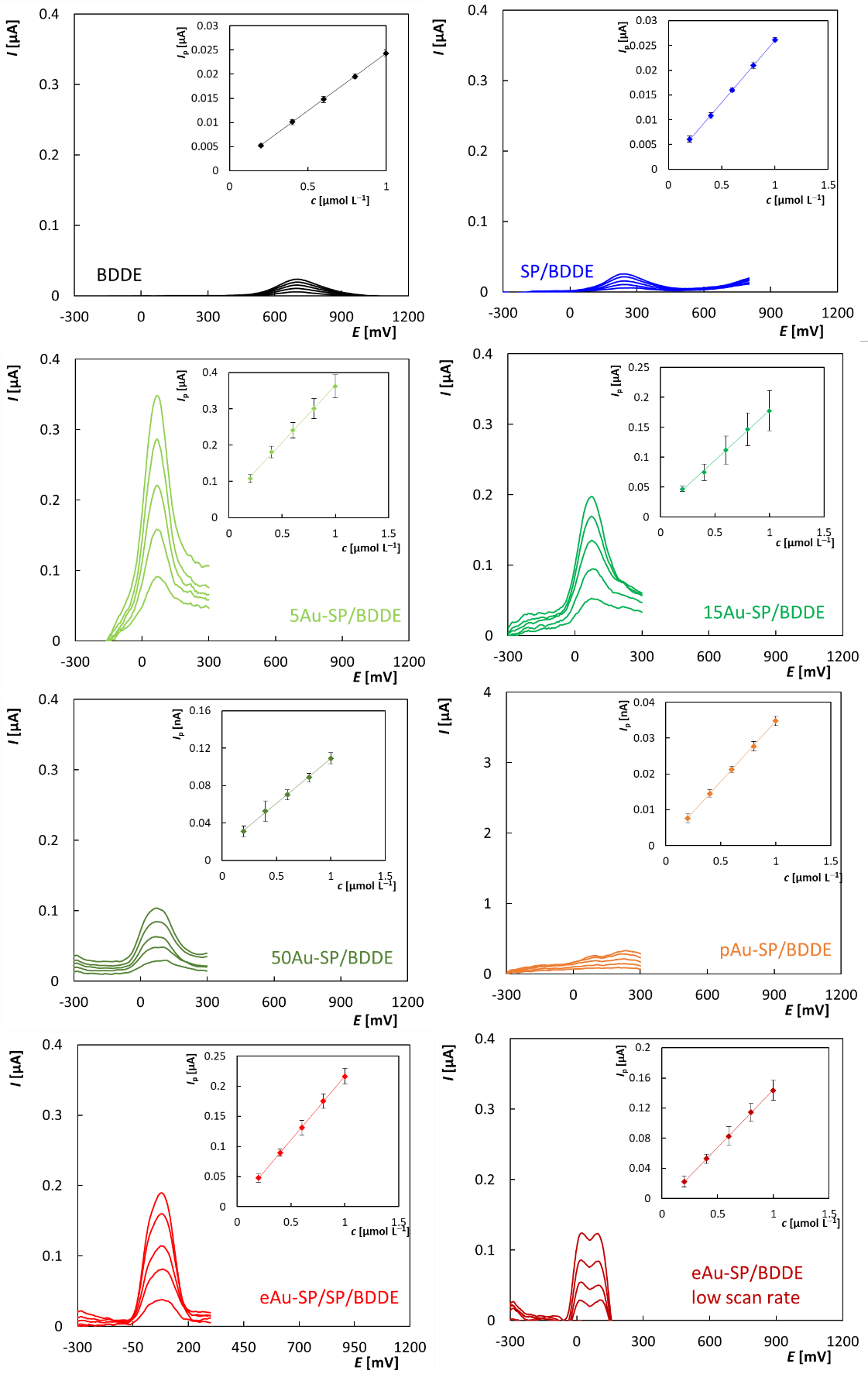


**Figure S15** SW voltammograms of DA recorded on a tested sensor depending on the concentration and the corresponding dependences of *I*_p_ on *c*; electrolyte – BRB (pH 5.5), *c*(DA) = 0.2-1 μmol L^−1^, ν = 50 mV s^–1^, *A* = 80 mV, *f* = 10 Hz.

**Table S7** Comparison of the obtained results with those previously published for DA determination

| Electrode | Method | Electrolyte | *LOD*  [nmol L^−1^] | Ref. |
| --- | --- | --- | --- | --- |
| BDDE | DPV | ABS (pH 3) | 90 | ^4^ |
| AuNPs-BDDE | SWV | PBS (pH 7.4) | 100 | ^5^ |
| Au/pBDDE | DPV | 0.1 M HClO_4_ | 60 | ^6^ |
| Au/PE/PS-BDDE | CV | PBS (pH 7.2) | 800 | ^7^ |
| Au-C@Ni/BDDE | DPV | 0.1 M HClO_4_ | 15 | ^8^ |
| Au-PANI/BDDE | SWV | PBS (pH 7.0) | 30 | ^9^ |
| GC/NiOx-Au | SWV | PBS (pH 7) | 79 | ^10^ |
| graphite–polyurethane | DPV | PBS (pH 7) | 15.5 | ^11^ |
| poly-taurine/AuNP/GCE | DPV | PBS (pH 7.38) | 160 | ^12^ |
| Au NP@3D GR | DPV | PBS (pH 7) | 100 | ^13^ |
| ErGO-AuNP/ITO | DPV | PBS (pH 7.4) | 15 | ^14^ |
| 3D-MoS_2_/rGO/Au@GCE | DPV | PBS (pH 7) | 110 | ^15^ |
| AuNPs/GCE | DPV | PBS (pH 7.2) | 130 | ^16^ |
| CoS_2_/IL-GN/GCE | DPV | PBS (pH 7.4) | 40 | ^17^ |
| rGO-Mn_3_O_4_/ Nafion-Au/GC | AM | PBS (pH 4.5) | 250 | ^18^ |
| MWCNT/PSVM/Au/GCE | DPV | PBS (pH 7) | 56 | ^19^ |
| Au/MWNTs/Nafion/GCE | DPASV | PBS (pH 6) | 40 | ^20^ |
| Au/ATP-ABA/GCE | DPV | PBS (pH 6) | 9200 | ^21^ |
| BDDE | SWV | BRB (pH 5.5) | 62.2 | in this work |
| SP/BDDE | SWV | BRB (pH 5.5) | 77.3 | in this work |
| 5Au-SP/BDDE | SWV | BRB (pH 5.5) | 2.5 | in this work |
| 15Au-SP/BDDE | SWV | BRB (pH 5.5) | 9.3 | in this work |
| 50Au-SP/BDDE | SWV | BRB (pH 5.5) | 36.6 | in this work |
| pAu-SP/BDDE | SWV | BRB (pH 5.5) | 13.4 | in this work |
| eAu-SP/BDDE | SWV | BRB (pH 5.5) | 12.8 | in this work |
| eAu-SP/BDDE (5 mV s^–1^) | SWV | BRB (pH 5.5) | 9.9 | in this work |

PE – polyelectrolyte, PS – polystyrene, Au – gold, pBDD – porous boron-doped diamond electrode, Au-C@Ni – gold nanoparticles and graphite-coated nickel nanoparticles, PANI – polyaniline, AM – amperometry, Au NP@3D GR – gold nanoparticles and 3-dimensional graphene; ErGO-AuNP/ITO – graphene oxide and gold nanoparticles on an indium tin oxide electrode; 3D-MoS_2_/rGO/Au@GCE – gold nanoparticles on a 3D-network consisting of a MoS_2_/rGO nanocomposite; MWCNT/PSVM/Au/GCE – carbon nanotubes and gold nanoparticles in an amphiphilic copolymer; Au/ATP-ABA/GCE – attachment of gold nanoparticles to glassy carbon electrode

**
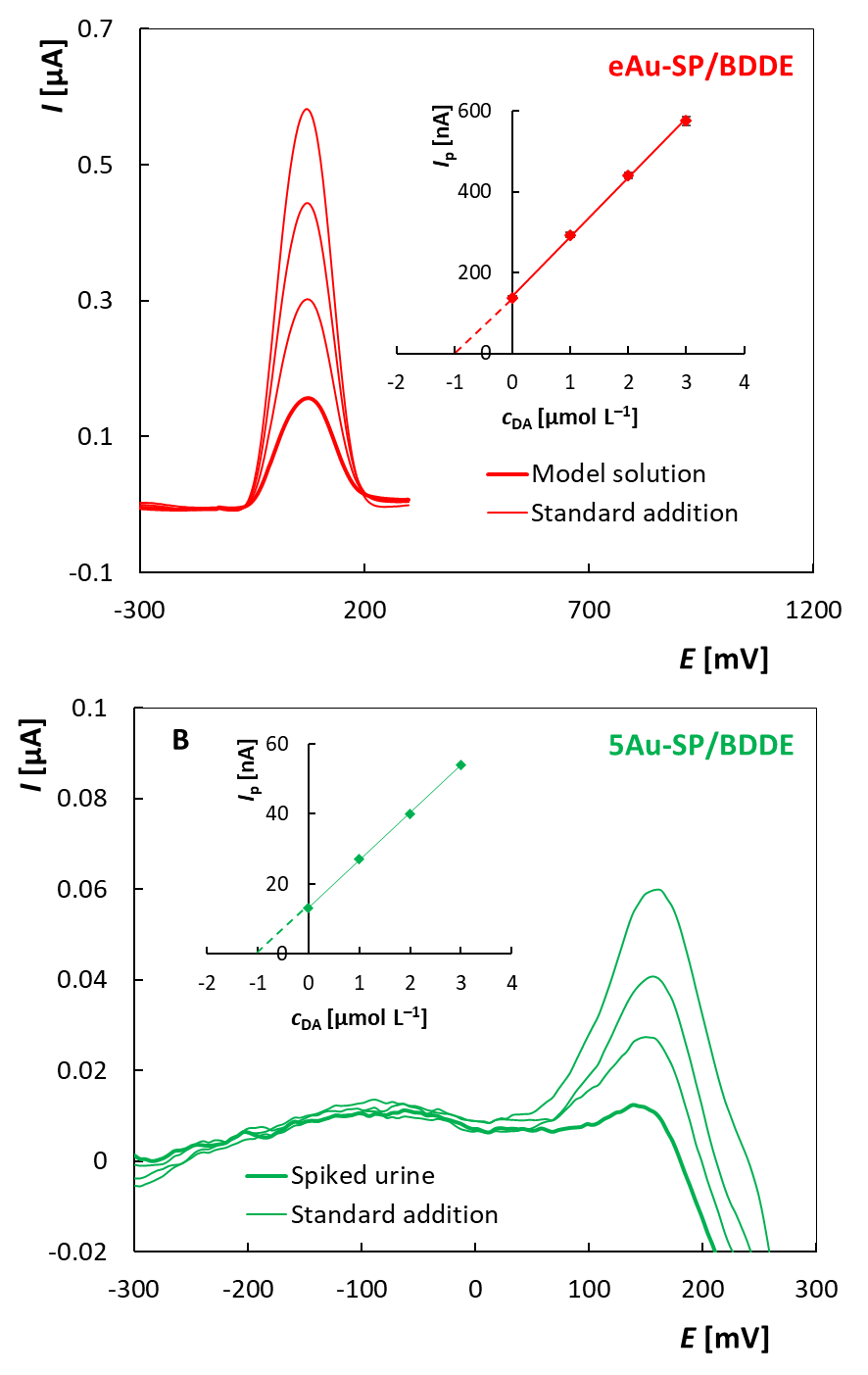
**

**Figure S16** Analysis of model solution using eAu-SP/BDDE (A) and of spiked urine using 5Au-SP/BDDE (B) with graphic evaluation of standard addition method; electrolyte – BRB (pH 5.5), *ν* = 50 mV s^–1^, *A* = 80 mV, *f* = 10 Hz.


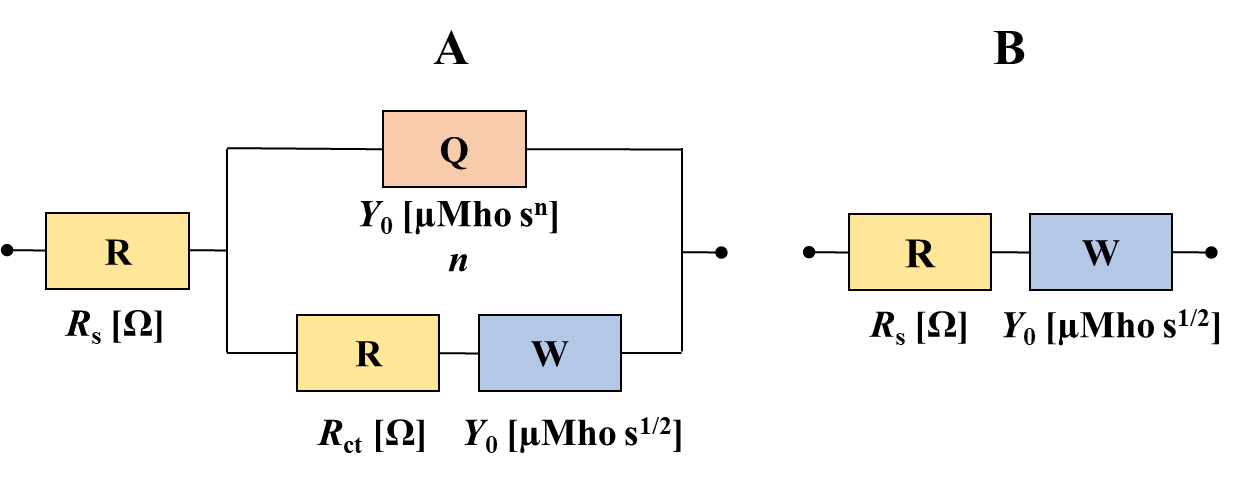


**Figure S17** Used electrical equivalent circuit (EEC) R(Q/[RW]) for the redox markers [Fe(CN)_6_]^4−/3−^ and DA (A) RW for the redox marker [Ru(NH_3_)_6_]^2+/3+^ (B). R – resistance, W – Warburg impedance, Q – constant phase element, *R*_s_ – serial resistance, *R*_ct_ – charge transfer resistance, *Y*_0_ – admittance, *n* – exponent of constant phase element.

**References**

1. Moore, W. M. & Codella, P. J. Oxidation of silver films by atomic oxygen. *J. Phys. Chem.* **92**, 4421–4426 (1988).

2. Pfeifer, R. *et al.* Generation of Nanoporous Diamond Electrodes Fabricated by a Low-Cost Process at Moderate Temperatures. *ACS Appl. Eng. Mater.* **1**, 1446–1454 (2023).

3. Breczko, J., Plonska-Brzezinska, M. E. & Echegoyen, L. Electrochemical oxidation and determination of dopamine in the presence of uric and ascorbic acids using a carbon nano-onion and poly(diallyldimethylammonium chloride) composite. *Electrochim. Acta* **72**, 61–67 (2012).

4. Sochr, J., Cinkova, K. & Svorc. Electrochemical Behaviour Study and Sensitive Determination of Dopamine on Cathodically Pretreated Boron-doped Diamond Electrode. *Austin. J. Anal. Pharm. Chem.* **1**, (2014).

5. Weng, J. *et al.* Gold-Cluster Sensors Formed Electrochemically at Boron-Doped-Diamond Electrodes: Detection of Dopamine in the Presence of Ascorbic Acid and Thiols. *Adv. Funct. Mater.* **15**, 639–647 (2005).

6. Mei, X. *et al.* Long-term stability of Au nanoparticle-anchored porous boron-doped diamond hybrid electrode for enhanced dopamine detection. *Electrochim. Acta* **271**, 84–91 (2018).

7. Wei, M. *et al.* Selective Determination of Dopamine on a Boron-Doped Diamond Electrode Modified with Gold Nanoparticle/Polyelectrolyte-coated Polystyrene Colloids. *Adv. Funct. Mater.* **18**, 1414–1421 (2008).

8. Li, H. *et al.* High-sensitivity, selective determination of dopamine using bimetallic nanoparticles modified boron-doped diamond electrode with anodic polarization treatment. *J. Mater. Sci.* **56**, 4700–4715 (2021).

9. Song, M.-J., Lee, S.-K., Kim, J.-H. & Lim, D.-S. Dopamine Sensor Based on a Boron-Doped Diamond Electrode Modified with a Polyaniline/Au Nanocomposites in the Presence of Ascorbic Acid. *Anal. Sci.* **28**, 583–587 (2012).

10. Al-thagafi, Z. T. & Awad, M. I. Voltammetric analysis of Dopamine in the Presence of Large Concentration of Ascorbic Acid at Nickel Oxide-Gold Nanoparticles Binary Electrocatalyst. *Int. J. Electrochem. Sci.* 5860–5877 (2020) doi:10.20964/2020.06.56.

11. Cervini, P., Mattioli, I. A. & Cavalheiro, É. T. G. Developing a screen-printed graphite–polyurethane composite electrode modified with gold nanoparticles for the voltammetric determination of dopamine. *RSC Adv.* **9**, 42306–42315 (2019).

12. Zhang, B. *et al.* In-situ graft-crosslinked gold nanoparticles with high-density surface defects and coated with a polytaurine membrane for the voltammetric determination of dopamine. *Microchim. Acta* **186**, 746 (2019).

13. Wang, Z. *et al.* Gold nanoparticles anchored onto three-dimensional graphene: simultaneous voltammetric determination of dopamine and uric acid. *Microchim. Acta* **186**, 573 (2019).

14. Huang, X., Shi, W., Bao, N., Yu, C. & Gu, H. Electrochemically reduced graphene oxide and gold nanoparticles on an indium tin oxide electrode for voltammetric sensing of dopamine. *Microchim. Acta* **186**, 310 (2019).

15. Zhao, Y. *et al.* In-situ growth of gold nanoparticles on a 3D-network consisting of a MoS2/rGO nanocomposite for simultaneous voltammetric determination of ascorbic acid, dopamine and uric acid. *Microchim. Acta* **186**, 92 (2019).

16. Zhao, H. *et al.* Additive-Free Gold Nanoparticles Induced by Gamma Ray Irradiation for Voltammetric Detection of Dopamine. *J. Nanosci. Nanotechnol.* **18**, 4495–4500 (2018).

17. Zhuang, X., Chen, D., Zhang, S., Luan, F. & Chen, L. Reduced graphene oxide functionalized with a CoS2/ionic liquid composite and decorated with gold nanoparticles for voltammetric sensing of dopamine. *Microchim. Acta* **185**, 166 (2018).

18. Yao, Z. *et al.* Voltammetric dopamine sensor based on a gold electrode modified with reduced graphene oxide and Mn3O4 on gold nanoparticles. *Microchim. Acta* **184**, 2081–2088 (2017).

19. Liu, J. *et al.* A nanocomposite consisting of carbon nanotubes and gold nanoparticles in an amphiphilic copolymer for voltammetric determination of dopamine, paracetamol and uric acid. *Microchim. Acta* **184**, 1739–1745 (2017).

20. Yang, S. *et al.* Immobilization of gold nanoparticles on multi-wall carbon nanotubes as an enhanced material for selective voltammetric determination of dopamine. *Sens. Actuators B Chem.* **178**, 217–221 (2013).

21. Zhang, L. & Jiang, X. Attachment of gold nanoparticles to glassy carbon electrode and its application for the voltammetric resolution of ascorbic acid and dopamine. *J. Electroanal. Chem.* **583**, 292–299 (2005).
